# High-Impedance Nonlinear Metasurface Arrays with Self-Decoupling for Modular and Wearable Magnetic Resonance Imaging

**DOI:** 10.34133/research.1057

**Published:** 2025-12-22

**Authors:** Enhua Xiao, Qiaoyan Chen, Shahzeb Hayat, Hao Li, Wenhao Liao, Peiyu He, Zhiguang Mo, Peng Cao, Xiaoliang Zhang, Dong Liang, Xin Liu, Hairong Zheng, Ye Li

**Affiliations:** ^1^ Lauterbur Imaging Research Center, Shenzhen Institutes of Advanced Technology, Chinese Academy of Sciences, Shenzhen 518055, China.; ^2^ Key Laboratory for Magnetic Resonance and Multimodality Imaging of Guangdong Province, Shenzhen 518055, China.; ^3^ Institute of Science and Technology for Brain-Inspired Intelligence, Fudan University, Shanghai 201203, China.; ^4^ Department of Diagnostic Radiology, The University of Hong Kong, Hong Kong, China.; ^5^ Department of Biomedical Engineering, State University of New York at Buffalo, Buffalo, NY 14260, USA.

## Abstract

Magnetic resonance imaging (MRI) signal acquisition relies heavily on radio frequency (RF) coils, which play a critical role in obtaining high-quality images. However, traditional RF coils are often rigid and bulky and require complex decoupling mechanisms, limiting their adaptability and effectiveness for anatomically curved or dynamic regions. In this study, we present a novel high-impedance nonlinear metasurface (HINM) coil based on a “building bricks” concept to overcome these limitations. The HINM coil features a lightweight, flexible, wireless, and compact design that enables direct attachment to various anatomical regions, providing localized signal enhancement without the need for intricate adjustments for decoupling. This innovative approach utilized a flexible coaxial cable with a shielded design, ensuring stable frequency characteristics under bending, stretching, and dynamic conditions. Furthermore, the HINM coil with passive detuning did not alter the RF transmit field distribution while improving the signal-to-noise ratio (SNR) up to 87% in the surface region, as demonstrated by the phantom studies. In the knee and hand imaging, the SNR in the joint and finger areas was also doubled when using the HINM arrays. Enhanced image quality was achieved, and more subtle blood vessels were revealed in the hand vascular imaging with the use of the HINM arrays in combination with the commercial RF coils. Additionally, the HINM arrays were applied for the knee and hand imaging at 0.5 T, which achieved up to 74% SNR improvement, demonstrating its effectiveness for both low-field and ultrahigh-field MRI. By offering a versatile and adaptable solution, the HINM coil demonstrates its potential to transform MRI coil designs, particularly for imaging anatomically complex and dynamic environments.

## Introduction

Magnetic resonance imaging (MRI) has become essential in modern healthcare, providing noninvasive, detailed images that greatly enhance the ability to diagnose, plan treatment, and monitor therapy responses for various diseases [1–4]. The performance and image quality of the MRI, especially in terms of signal-to-noise ratio (SNR), is mainly determined by the radio frequency (RF) receiver coils [5–9]. The traditional receiver coils are reliable but are renowned for their bulky, fixed, and rigid configuration, causing discomfort to patients and compromising the signal’s sensitivity in certain situations. Furthermore, these coil designs focus mainly on a specific part of the body to ensure high SNR, necessitating separate coils to accommodate different anatomical structures such as the hand, knee, foot/ankle, and pelvis, which increases the overall hardware cost.

Wearable technologies, including MRI receiver coils, have gained attention in recent years due to their form-fitting and flexible designs [10–21]. Several methods are used in wearable MRI coils, such as using conductive threads [10,11], electro-textile [12], conductive elastomers [13], liquid metal [14,15], screen-printed traces [16], and coaxial cable [17–20]. These methods enhanced patient comfort and achieved comparable SNR by conforming more closely to the average anatomy than the dedicated traditional coils. However, these wearable coils required further improvement in SNR. Moreover, these wearable MRI coils often include non-electromagnetic components, such as adapters, cable traps, feeding boards, and decoupling preamplifiers, increasing their bulk and cost and requiring careful handling during MRI scans.

Alternatively, the electromagnetic metamaterials or metasurface structures are inexpensive and compact designs that improve SNR in combination with the commercial receiver coils [22–36]. In such setting, metasurface structure and commercial receiver coils are inductively coupled, eliminating the need for additional components such as baluns, preamplifiers, coil plugs, and hazardous cable effects that are expensive and tailor-made for the receiver coils. The principle of these electromagnetic structures is to control the propagation path and field distribution of electromagnetic waves by using the interaction effect between electromagnetic waves and metal or dielectric elements and the coupling effect between elements [22]. Additionally, recent advances in electromagnetic materials, such as fractal left-handed superlenses and LPDA-inspired metapyramids, show how the combination of geometric and material design can enhance magnetic field control, offering enhanced resolution, broadband, and angle-insensitive performance [23]. Furthermore, electromagnetic metamaterials or metasurface structures used for magnetic resonance SNR enhancement can generally be categorized into 2 types: (a) linear metamaterial structures, which simultaneously enhance both the RF transmit and receive magnetic fields [22–28]; and (b) nonlinear metasurface structures, which selectively enhance the RF receive magnetic field without interfering with the RF transmit field [29–41].

For the linear structures, they can be divided into plane metasurfaces [24–27] and volume metamaterials [28]. Planar metasurface structures include Swiss coil arrays [24], negative permeability split ring arrays [25], and wire arrays [26]. However, these structures did not perform well in terms of magnetic field uniformity, especially in the direction perpendicular to the structures. The nonuniform distribution of the RF magnetic field would change the image contrast, increase the artifacts, and even lead to the possibility of misdiagnosis. Although the magnetic field uniformity of volume metamaterials was greatly improved than that of planar metasurface structures [28], they were usually particularly large and occupied more imaging space, which was impractical for the clinical application. More recently, a flexible metasurface of spiral quartet arrays was proposed to enable efficient imaging of body parts with varying curvatures [27], which demonstrated good application potential. However, the linear structures had an enhanced effect on the RF transmitting magnetic field, which would lead to an increase in RF energy deposition power, resulting in a potential safety problem.

For the nonlinear metasurface structures, Zhao et al. [31] and Li et al. [32] developed a nonlinear metasurface structure in the shape of a solenoid array, which did not take into account the decoupling between elements, resulting in nonuniform images. In order to achieve uniform images, Chi et al. [33] developed a cylindrical shape nonlinear metasurface structure. In addition, Zhu et al. [34] also developed a nonlinear metasurface structure with the shape of a Litzcage coil, which can also achieve uniform images. However, the above solenoid arrays and cylindrical shape nonlinear metasurface structures were rigid and could not be well adapted to the imaging subjects, which were limited in different applications. In order to achieve better conformal flexibility, researchers developed nonlinear metasurface structures with flexible planar solenoid arrays [35], coaxial line arrays [36,37], and planar loops [38–41], which can be well adapted to imaging different subjects with greater flexibility. However, electromagnetic decoupling is often not adequately addressed in the previously mentioned designs, especially those using nonlinear or distributed metasurfaces. When adjacent coil elements are placed in close proximity, physical inductive and capacitive coupling can occur. Specifically, the magnetic field generated by one element may induce unwanted currents in neighboring elements (mutual inductance) [42], while minimal spacing between elements can lead to capacitive crosstalk [43]. These effects distort the sensitivity profiles, resulting in spatially variable signals, reduced parallel imaging performance, and image artifacts [44,45]. This issue becomes particularly critical in conformal or wearable applications [46], where the coil arrays must operate in close-packed and dynamic configurations.

Moreover, most existing metamaterial-based coils demonstrating SNR improvement have been evaluated at low field, 1.5- and 3-T MRI [47,48], where spatial resolution are relatively modest. At higher field strengths, such as 7 T [49], although the intrinsic SNR is higher, the RF wavelength becomes shorter, leading to pronounced transmit field (B_1_^+^) inhomogeneities, signal dropouts, nonuniform image quality [50–52], and narrow anatomical applicability—typically confined to brain or extremity imaging. In contrast, 5-T MRI [53,54] offers a promising middle ground, delivering enhanced image resolution and SNR compared to 3 T, while retaining broader anatomical coverage and whole-body imaging capabilities that are often not feasible at 7 T. Since the 5-T MRI system is still a relatively new device, many dedicated RF coils have not yet been developed or the performance of the existing RF coils remains suboptimal. Therefore, a universal nonlinear metasurface structure that can enhance imaging performance in combination with the existing RF coils for imaging various anatomical regions needs to be developed. This metasurface should be passive, flexible, and intrinsically decoupled. The high-impedance coil (HIC) design enables self-decoupling of array elements for RF coils, which can then be combined with a metasurface structure [17,55]. In the HIC design, one or multiple lumped capacitors were placed on top of coils to create a high input impedance, while the entire coil loop needed to be matched to 50 ohms in order to achieve signal transmission. Studies also showed that the coaxial substrate can be configured as HIC to achieve self-decoupling, using the loop of inner/outer conductors with open gaps to form resonators [17]. While traditional HICs use custom coaxial cables to achieve the resonant frequency and high-impedance characteristics, they typically require multiple components, such as baluns, matching circuits, decoupling circuits, and preamplifiers, increasing both system complexity and cost. Similarly, although flexible metasurfaces offer improved anatomical conformity, their resonance performance tends to degrade under mechanical deformation or dynamic movement, compromising imaging reliability.

In this study, we introduce a high-impedance nonlinear metasurface (HINM) coil, a novel, flexible, and modular platform that can achieve intrinsic decoupling without requiring any active circuitry for interchannel coupling. To the best of our knowledge, this is the first study to integrate the principles of both high-impedance and metasurface technologies into a single, fully passive, anatomically independent design for MRI signal enhancement. The HINM coil was based on a miniaturized coaxial architecture that introduced an open gap in the outer conductor-unlike conventional coaxial loops, where both inner and outer conductors formed a closed ring. This structural gap in combination with an inductor and a capacitor created a shielded resonant configuration that effectively suppressed electromagnetic interference, maintained resonance stability under bending or stretching, and enabled efficient signal detection. Furthermore, the HINM coil featured a passive switching mechanism and characterized by strong self-decoupling without external biasing or active matching, eliminating the need for external control circuitry. To enhance versatility and usability, the HINM coil was implemented using a modular “building brick” concept, allowing custom arrays to be easily assembled and directly conform to curved or dynamic body regions. Notably, the previous metasurface-based designs have often suffered from signal inhomogeneity and visible image gaps due to the nonoverlap or nondecoupling elements [32,37], and the designs in the literatures [32,37] in 2024 were replicated and compared with the proposed HINM, as shown in Figs. S1 to S3. In contrast, the proposed HINM design incorporated shielded elements to enhance resonance stability and image uniformity, improving both reliability and clinical applicability. Furthermore, Table S1 provides a comprehensive comparison of the HINM coil, evaluating not only its performance across different MRI field strengths but also key practical features such as flexibility, stability, conformability, bending and stretching capabilities, modularity, weight, and overall cost, highlighting its advantages over existing coil designs.

**Fig. 1.**
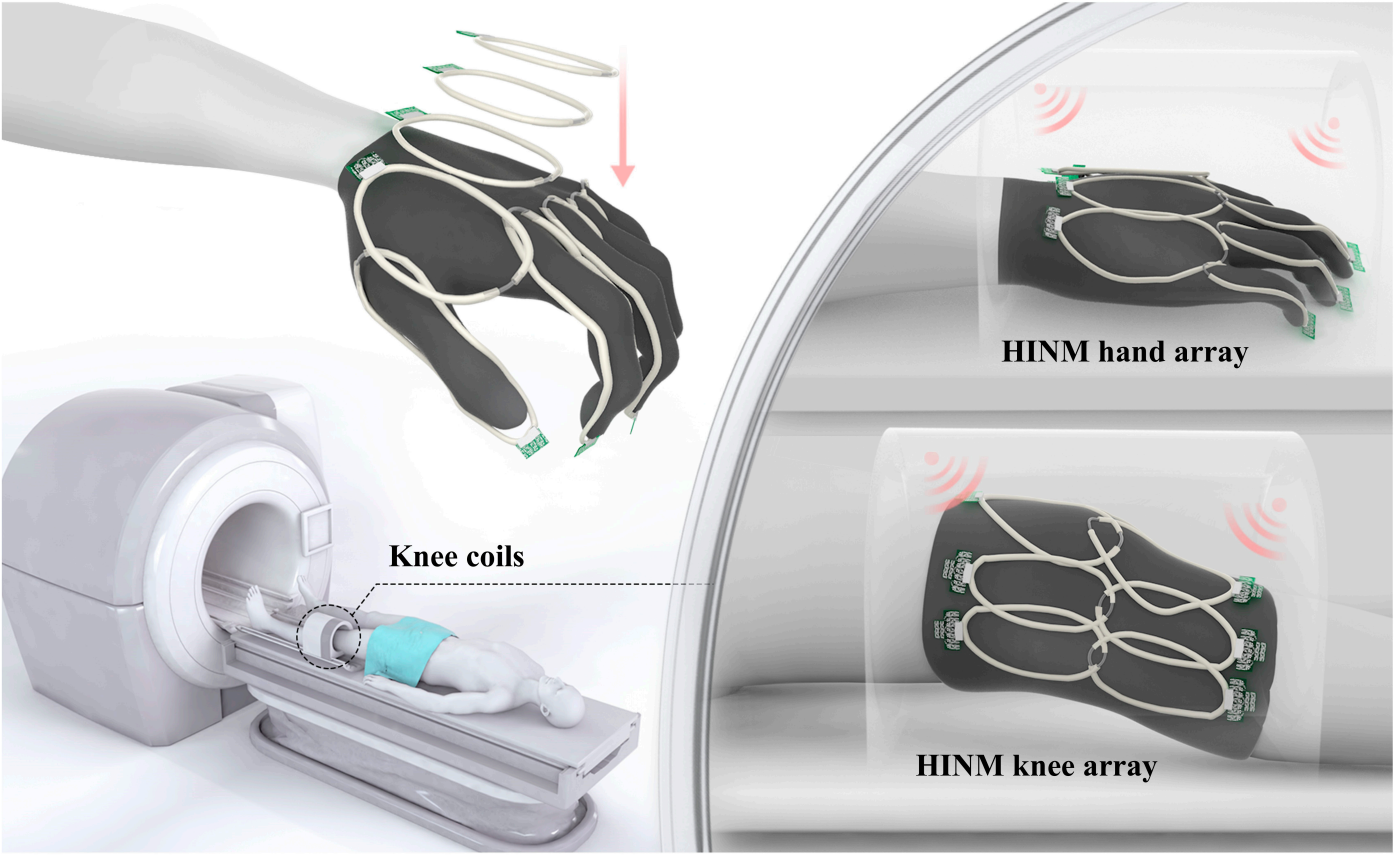
Illustration of the high-impedance nonlinear metasurface (HINM) arrays’ usage, demonstrating its application on the hand and knee.

As illustrated in Fig. 1, the HINM array demonstrates excellent anatomical adaptability and an ultralightweight form factor (only 3 g), ensuring patient comfort during scanning. The figure also highlights the ease with which flexible, modular arrays can be integrated into standard MRI workflows. Compared to the conventional rigid knee coils, the HINM system provides a more practical, comfortable, and patient-friendly solution for wearable MRI. The HINM arrays were tested on various anatomical regions such as the knee (flat and bent), hand (including fingers in different flexed postures), neck, and elbow (including bending). The coil maintained stability during bending up to 90° and stretching up to 80 mm, demonstrating consistent SNR improvement and reliability under various conditions. The HINM coil was further characterized through B_1_^+^ field interaction analysis, RF safety assessment, and temperature measurement. Importantly, the HINM was applied at 0.5 T for the knee and hand imaging, confirming that the design could substantially improve signal quality even at low-field strength. These results highlight the potential of the HINM arrays as a modular and efficient alternative to conventional coil design, paving the way for more customizable and user-friendly MRI solutions.

## Results

### Electromagnetic performance and bench test results

**Fig. 2.**
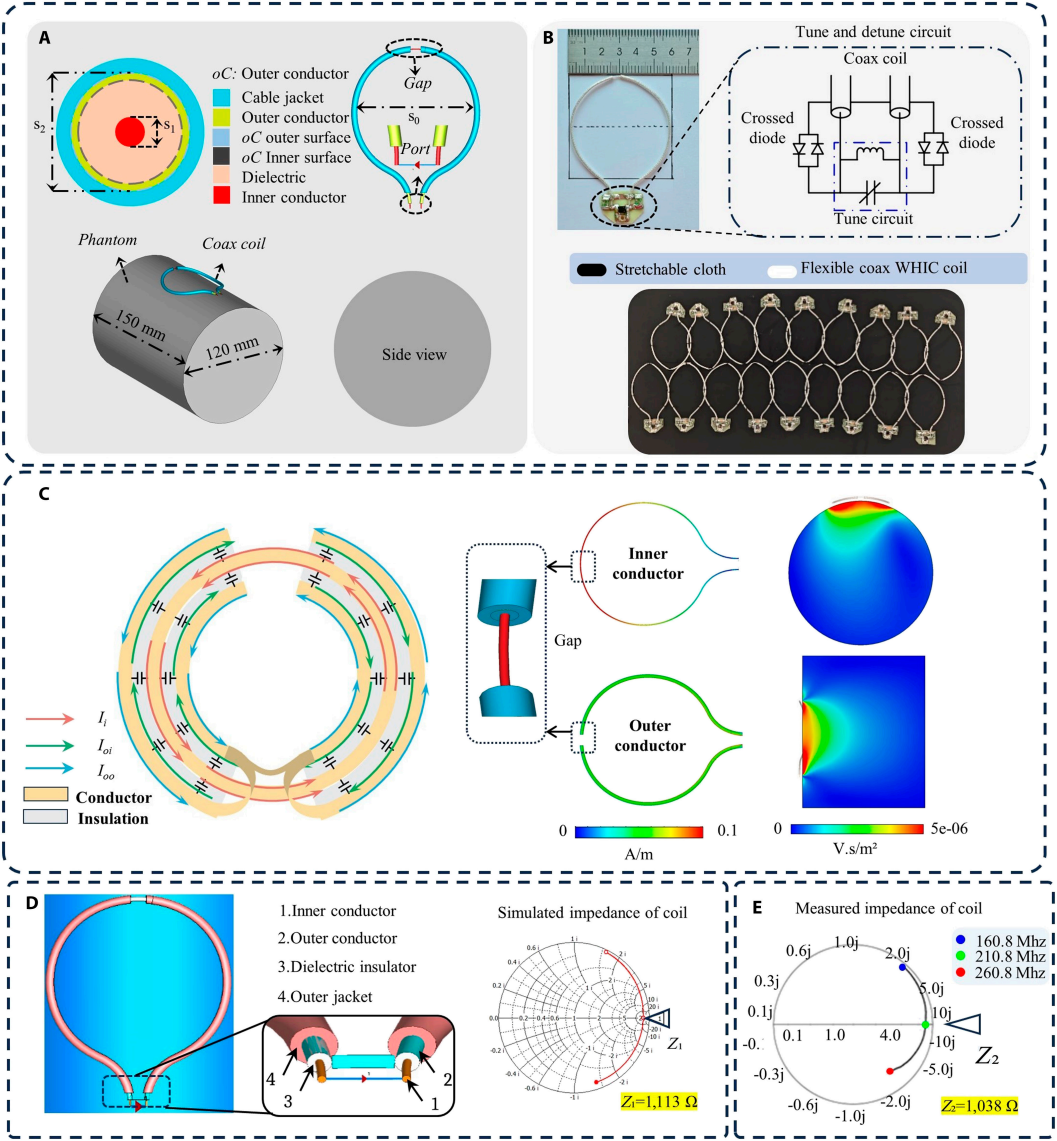
Geometry and simulation setup of the proposed HINM: (A) Geometry and cross-section overview of the HINM along with cylindrical phantom. (B) Ultralightweight fabricated prototype along with the tune/detune circuit. (C) Surface current distribution on the inner and outer conductors, and the magnetic field distribution in the coronal and sagittal planes. (D and E) Simulated and measured impedance of the HINM.

Figure 2 illustrates the geometry, fabricated prototype, and electromagnetic simulation results of the HINM coil. The HINM coil employed a coaxial cable structure with a strategically introduced gap in its outer conductor. This gap was pivotal in establishing a shielded configuration, facilitating effective decoupling from surrounding components and enabling efficient signal detection. The coaxial cable was composed of 3 separate conductors: the inner conductor (iC), the inner surface of the outer conductor (oCi), and the outer surface of the outer conductor (oCo). The sufficiently thick outer conductor can ensure isolation between the currents on oCi and oCo over a frequency range of 50 to 500 MHz, supported by the small skin depth relative to the shield thickness.

The electromagnetic simulations provide insights into the current distribution and the B_1_ magnetic field generated by the HINM coil. As shown in Fig. 2C, the current on the inner conductor was concentrated near the shield gap and was increased linearly along its length, a behavior characteristic of the self-decoupling design. On the outer conductor, the current distribution mirrored this behavior while remaining confined to its respective surface. This field separation resulted from the controlled capacitive coupling across the open gap, which localized both electric and magnetic fields. The shielded condition plays a critical role in suppressing mutual coupling. In the conventional copper wire coils, electromagnetic fields can easily induce unwanted currents in adjacent elements, leading to resonance shifts and signal distortion. In contrast, the HINM structure acts as an electromagnetic barrier, effectively mitigating such interactions. The decoupling performance in the simulation is shown in Fig. S4. The capacitive gap not only enables resonance tuning but also contributes to intrinsic decoupling by confining electric fields and limiting crosstalk between elements. As a result, the HINM coil operates independently, even in tightly packed array configurations, without requiring additional decoupling circuitry.

The B_1_ magnetic field, visualized in the coronal and sagittal planes (Fig. 2C), was concentrated near the shield gap. This localized enhancement of the B_1_ field can ensure uniform signal transmission and reception, particularly in the regions close to the coil. This uniformity is especially beneficial for imaging curved anatomical structures, which is critical for achieving high-quality MRI scans. The robustness of the B_1_ field distribution was preserved even under mechanical bending and deformation, highlighting the HINM’s adaptability to complex geometries.

Figure 2D and E show reasonable agreement between the simulated and measured impedance of the HINM. The impedance magnitude remained consistently high at approximately 1,038 ohms. Since the wireless coils only need to be tuned without matching, this is a unique high-impedance characteristic of the wireless coils. When this configuration is used as a wired RF coil, an additional matching circuit is required, and it should be matched to 50 ohms to achieve wired signal transmission.

**Fig. 3.**
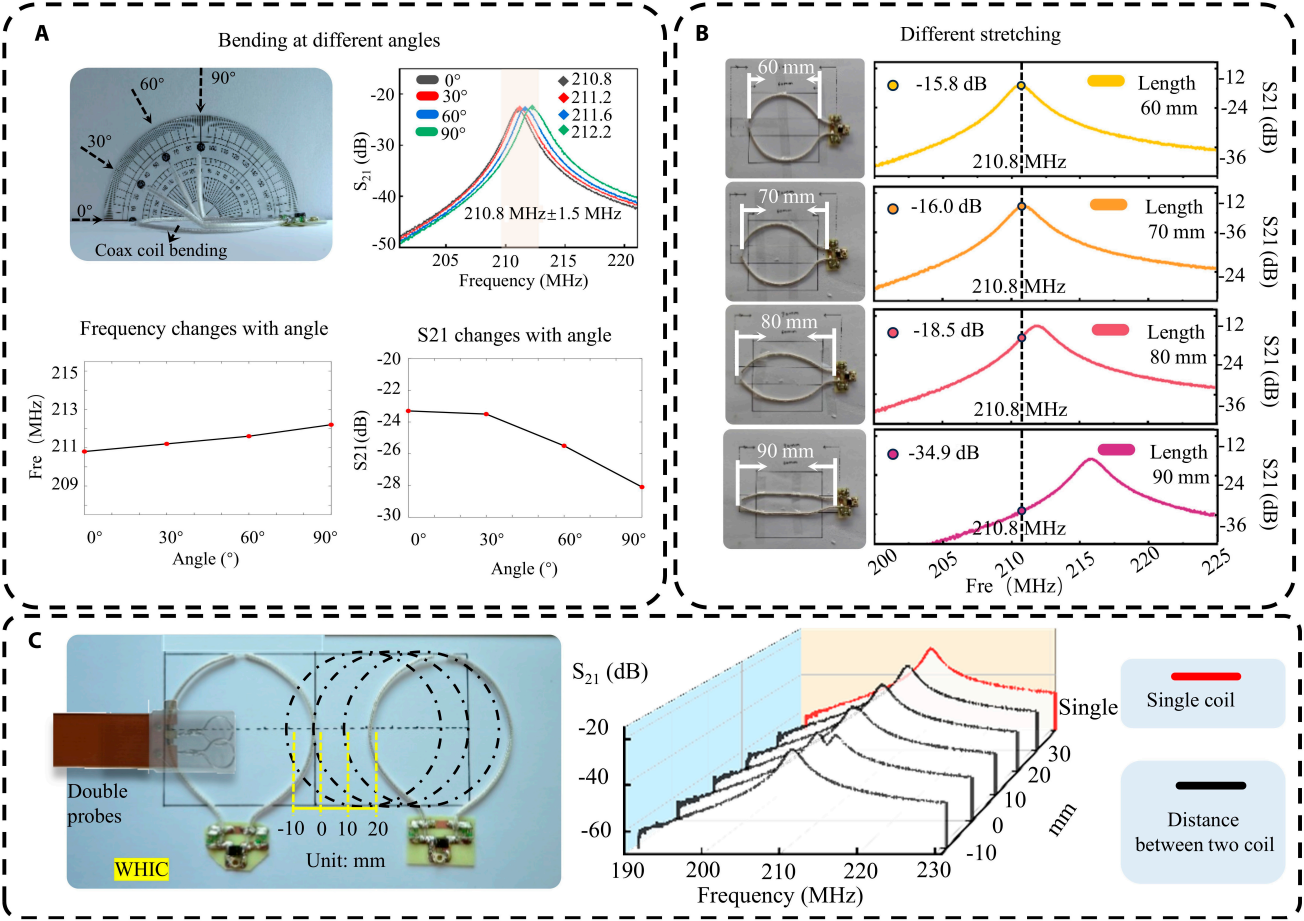
Bench test performance of the proposed HINM at various scenarios. (A) Resonance frequency of the HINM at various bending angles. (B) Resonance frequency performance of the HINM at different stretched lengths. (C) Decoupling performance at various distance between the 2 elements.

The electromagnetic performance of the HINM coil was evaluated under controlled bending and stretching deformations in order to assess its mechanical tolerance. The coil was incrementally bent from 0° to 90° in 30° steps, and stretched from 60 to 90 mm in 10-mm increments, as shown in Fig. 3A and B. The coaxial structure with a continuous inner conductor and an outer shield featuring a small gap ensured minimal changes in the electromagnetic boundary conditions during deformation. During bending, the radius of curvature introduced minor changes in the physical spacing between the inner conductor and the outer shield. However, due to the cylindrical symmetry and the uniform dielectric environment, the per-unit-length inductance and capacitance of the coaxial transmission line remained nearly constant.

As shown in Fig. 3A, increasing the bend angle from 0° to 90° resulted in only a slight shift in the resonance frequency, from 210.8 to 212.2 MHz. While the S_21_ curve exhibited increased signal loss at 90°, the overall performance of the HINM coil remained satisfactory. Similarly, stretching the coil from 60 to 80 mm produced only minimal changes in both resonance frequency and S_21_ magnitude. However, when stretched further to 90 mm, the S_21_ magnitude showed a more pronounced deviation, as illustrated in Fig. 3B. In spite of the minimal resonance shift, excessive stretching may introduce tension-induced asymmetries and impair mechanical integrity, ultimately compromising long-term stability. Overall, HINM coils exhibited remarkable mechanical and electromagnetic stability up to 90° and up to 80 mm, but performance at more extreme stretching (e.g., 90 mm) is degraded.

Moreover, we conducted cyclic bending tests up to 1,000 times, simulating typical wearable stress conditions, as shown in Fig. S5. The coil was repeatedly bent and returned to its original shape, and the S-parameters were remeasured. It is confirmed that the resonance frequency remained at approximately 210.8 MHz throughout the test, with minor fluctuations of ±0.2 MHz. The S_21_ amplitude exhibited minimal variation as well, staying within a narrow range between −15.25 and −16.11 dB. The coil showed no visible mechanical damage or performance degradation after prolonged mechanical stress, indicating its ability to remain tuned and transmit. Furthermore, the *Q* factor showed only negligible changes, confirming that the characteristics of HINM remained intact over time. Therefore, the HINM coil is well-suited for long-term wearable applications involving repeated bending and handling.

Figure 3C depicts the decoupling performance of the HINM coil, evaluated by analyzing the resonance frequency changes as a function of the separation distance between the 2 coil elements. The results indicate that the HINM coil exhibits a strong inherent decoupling feature; minimal coupling was observed across different distances. This performance was attributed to the shielded design, which suppresses mutual inductive coupling between neighboring elements. However, a distinct dip was observed near 210.8 MHz when the distance between adjacent HINM elements was reduced to zero, as shown in Fig. 3C. This behavior was caused by a mutual coupling between the elements when they were placed too close together. As shown in Fig. S6, a tightly packed configuration supported anti-symmetric current patterns, meaning that current flowed on neighboring elements in opposite directions. Due to destructive interference in the shared magnetic field region, the resonance at that frequency was effectively suppressed. Additionally, the surface current at 210.8 MHz clearly illustrates the out-of-phase distribution causing the dip. It is crucial to maintain optimal performance when designing tightly packed or modular coil arrays by ensuring proper element spacing. The results highlight the importance of maintaining optimal element spacing in coil arrays that are closely packed or modular. The HINM coil demonstrates robust intrinsic decoupling, which ensures that each coil element operates independently despite its proximity, even in high-density array applications.

### Imaging performance

**Fig. 4.**
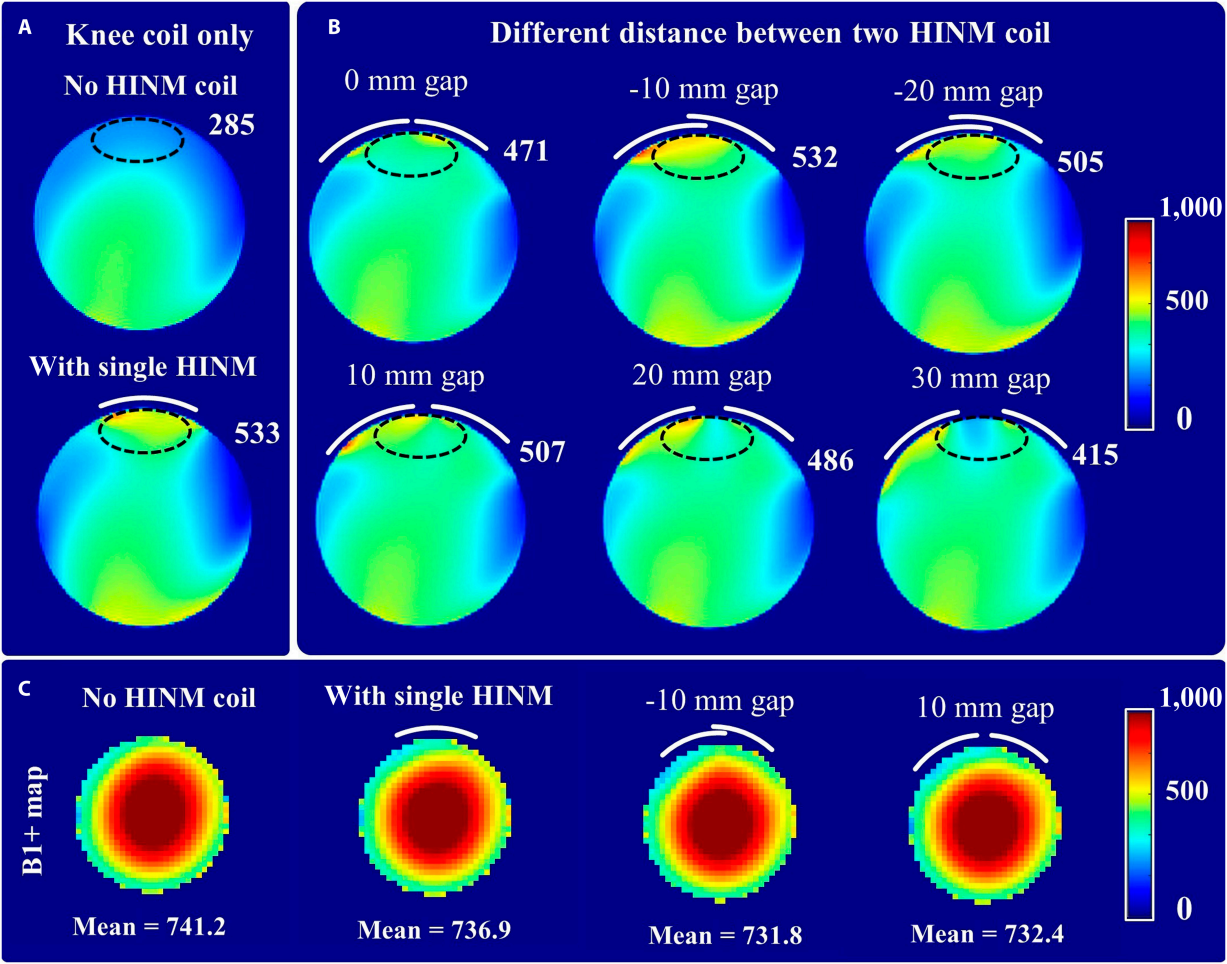
Comparison of signal-to-noise ratio (SNR) for a 24-channel knee coil, combined with a single-channel HINM coil, and 2 HINM coils at different distances. (A) The SNR for the knee coil alone and with the single-channel HINM coil. (B) The SNR for the knee coil with the 2 HINM coils at different distances. (C) The transmit field (B_1_^+^) maps of the knee coil and its integration with different HINM coils.

Figure 4 demonstrates the capability of the HINM coils to enhance the SNR in knee imaging, highlighting its potential to surpass the limitations of conventional receiver coils. The comparison in Fig. 4A shows the SNR performance of a standard 24-channel knee coil used independently and in combination with a single-channel HINM coil. The results reveal a pronounced SNR improvement of 87% in the regions proximal to the HINM coil. This enhancement was attributed to the HINM’s ability to optimize the magnetic field distribution near its surface, providing a marked boost in signal detection sensitivity.

Figure 4B shows the impact of varying the separation distance between 2 HINM coils. The results indicate stable and consistent improvement in SNR across different configurations, ranging from 46% to 87%. The inherent decoupling characteristics of the HINM coil, enabled by its shielded design, mitigated mutual coupling effects, even at reduced separations. This property ensures that the HINM coil can be effectively integrated into multielement systems without compromising the performance of elements or the overall imaging quality.

The B_1_^+^ maps obtained with the HINM coils in combination with the commercial knee coil exhibited less than 3% of the signal strength as compared to that acquired with the only commercial knee coil, as shown in Fig. 4C. This indicates that the HINM coil can substantially enhance the receive field strength while having minimal impact on the transmit field. These eliminate the concerns regarding B_1_^+^ inhomogeneity or RF safety. Moreover, RF safety, such as specific absorption rate (SAR), is associated with the transmit field strength. The transmit field stability will help minimize localized heating and ensure patient safety during MRI procedures.

**Fig. 5.**
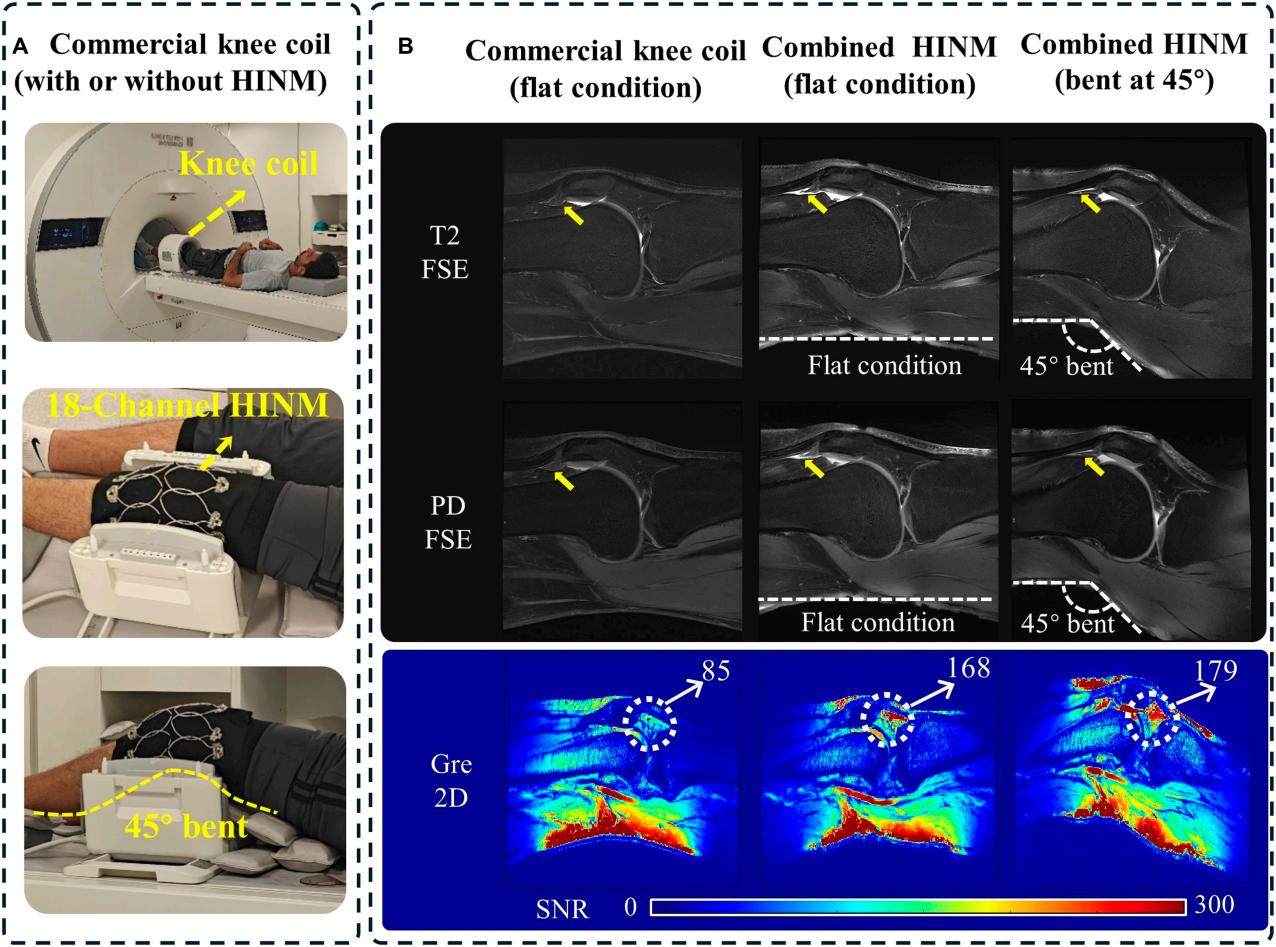
The performance of the 18-channel HINM array in a healthy volunteer subject. (A) The photographs of the in vivo measurement setup consisting of the 18-channel HINM array. (B) In vivo images of the knee with a resolution of 0.37 × 0.37 × 3 mm^3^ and the SNR maps using the commercial knee coil without and with the 18-channel HINM array.

Figure 5 demonstrates the performance of the 18-channel HINM array for knee imaging, highlighting its adaptability and high-resolution imaging capabilities. Figure 5A shows the experimental setups. The ultraflexible and lightweight HINM design allowed it to conform closely to anatomical surfaces, enabling optimal contact and improved signal reception. This close coupling ensured uniform proximity to the region of interest (ROI), optimizing electromagnetic coupling and facilitating high signal sensitivity. Figure 5B compares the MR images and the SNR maps acquired using the conventional knee coil versus those obtained with the commercial coil combined with the HINM array, under both flat and bent conditions. The HINM-enhanced images clearly demonstrate superior anatomical detail, particularly in bent positions, where the conventional coils often experience obvious performance degradation. This improvement was driven by the proposed HNIM design, which inherently suppresses mutual coupling between adjacent elements, preserving signal integrity and enabling dense array configurations without additional interelement decoupling circuitry. The flexible, modular structure of the HINM array allowed it to adapt to complex anatomical contours, further improving electromagnetic coupling and overall SNR. Compared to the conventional rigid or semirigid coils, the HINM arrays demonstrated superior performance, especially under dynamic conditions such as bending or slight patient movement.

Figure 5 also shows quantitative SNR maps, demonstrating a substantial improvement in SNR within the ROI, especially at the anterior horn of the lateral meniscus. The SNR increased by approximately 97.6% in the flat condition and 110.6% in the bent condition. Furthermore, PD-FSE imaging also yielded SNR enhancement in the ROI using the HINM array compared to the only commercial knee coil under both flat and bent configurations, which offered substantial improvements in anatomical visualization with 0.37 × 0.37 × 3 mm^3^ voxels.

**Fig. 6.**
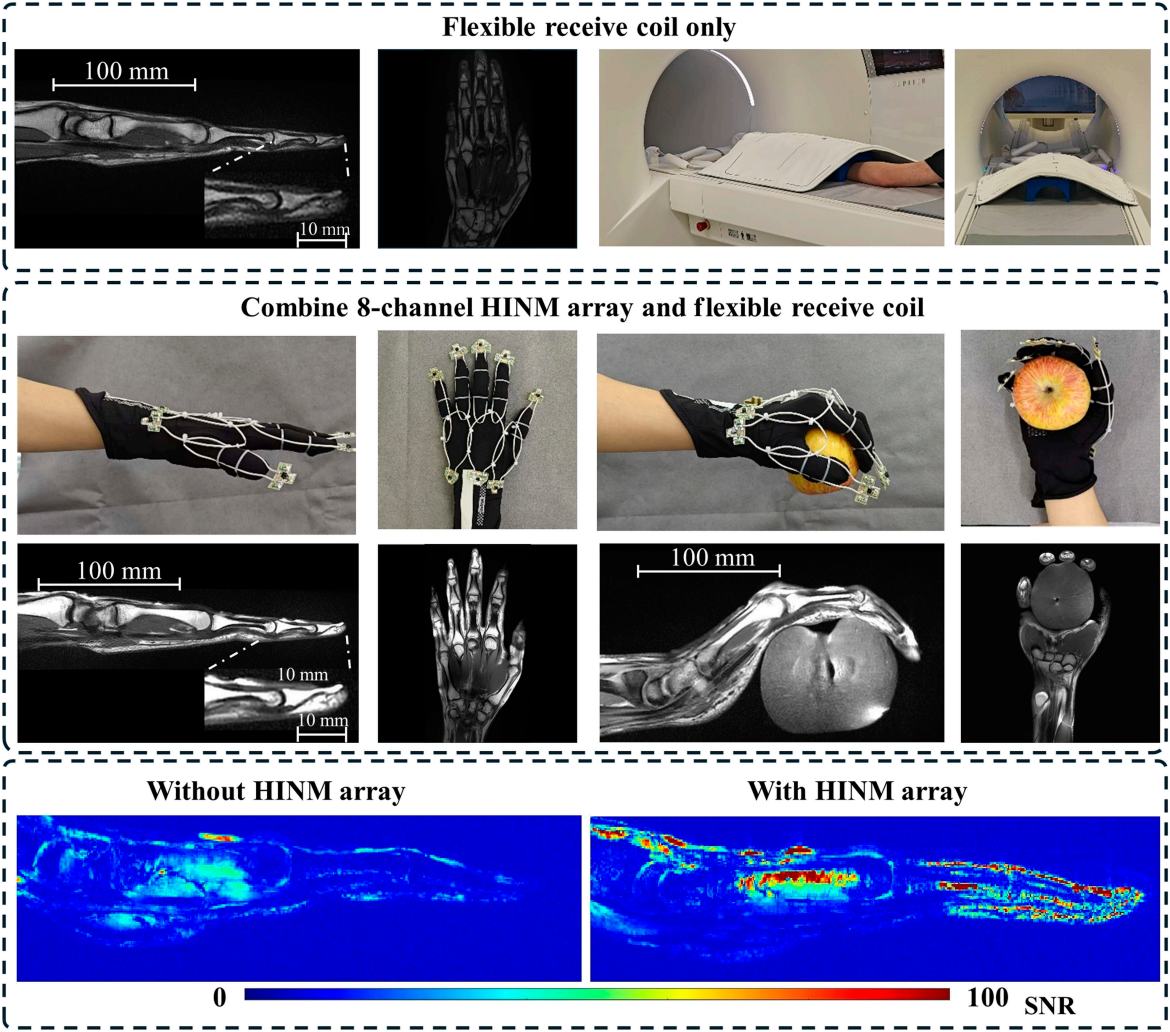
Performance of an 8-channel HINM array in a healthy volunteer subject. In vivo images of the hand with a resolution of 0.53 × 0.53 × 3 mm^3^ and the SNR maps using the commercial flexible receiver coil without and with the 8-channel HINM array.

Figure 6 illustrates the in vivo imaging performance of an 8-channel HINM array for hand imaging, highlighting its ability to achieve a high resolution of 0.26 × 0.26 × 2 mm^3^ with nearly double SNR compared to a commercial flexible receiver coil. The HINM’s shielded design and flexible structure enabled it to conform closely to the intricate contours of the hand, ensuring consistent proximity and optimized signal reception across the imaging region. This adaptability was particularly evident in the clear visualization of anatomical details such as bones, tendons, and joint spaces, even in areas with complex geometries. The HINM array achieved marked SNR improvement, providing sharper delineation of small structures and enhanced image quality as compared to the commercial flexible receiver coil. Despite pronounced curvature or varying finger positions, the HINM array was able to maintain stable resonance during imaging. The lightweight and flexible design of HINM also contributed to greater comfort and ease of use, making it especially suitable for imaging during hand motion or in anatomically complex regions. The HINM array not only achieves higher SNR and spatial resolution than the conventional coils but also offers significant advantages in flexibility and adaptability, allowing advanced imaging of complex and mobile anatomical regions like hands.

**Fig. 7.**
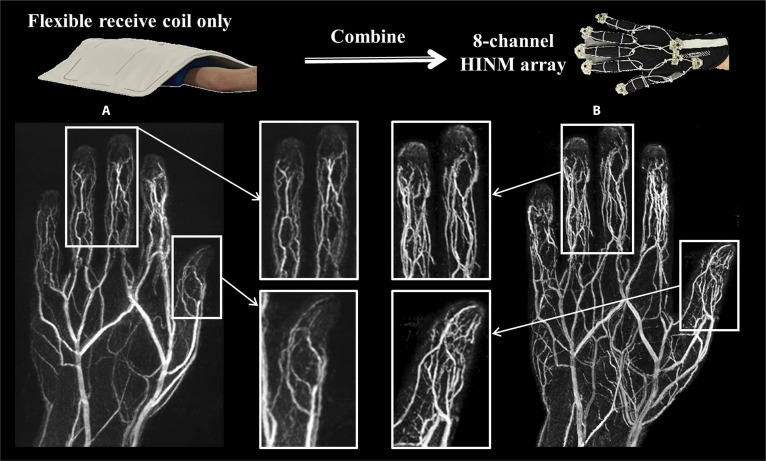
Hand vascular imaging with a resolution of 0.5 × 0.5 × 0.5 mm^3^. (A) Using the only commercial flexible receiver coil. (B) Using the commercial flexible receiver coil in combination with the 8-channel HINM array.

The 8-channel HINM array can also be applied in other clinical scenarios. For instance, when imaging the palmar vasculature, traditional methods typically rely on clinical standard contrast-enhanced angiography, which requires intravenous injection. This approach not only carries inherent risks but also fails to adequately visualize small vessels. Here, we employed a non-contrast-enhanced 3-dimensional (3D) phase-contrast MR angiography of the hand (voxel size: 0.5 mm isotropic; velocity encoding: 3 cm/s), presented as maximum-intensity projection images. The image in Fig. 7A was acquired using the commercial flexible receiver coil covering the dorsal side of the hand without the HINM array, visualizing dorsal veins but providing a limited depiction of the palmar arteries. The image in Fig. 7B was obtained using the commercial flexible receiver coil combined with the 8-channel HINM array covering the palmar side, substantially enhancing the visualization of palmar arteries and small vessels. This approach offers potential for future treatments of conditions like hand vascular malformations.

**Fig. 8.**
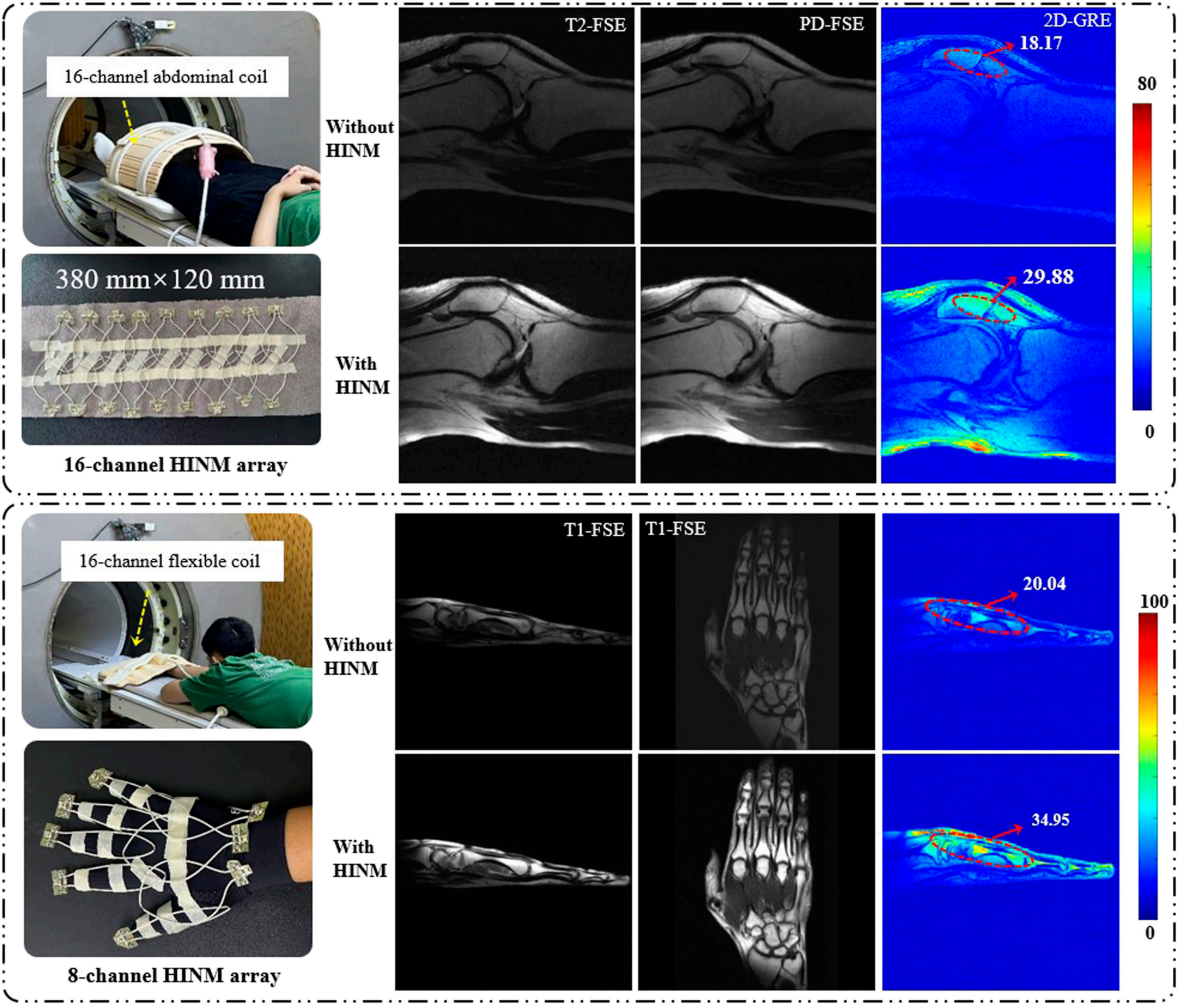
Low-field (0.5 T) MRI of the knee and hand using a commercial 16-channel abdomen coil and a 16-channel flexible coil with the HINM arrays.

Additionally, the HINM coil with the same size was tuned and matched at a lower Larmor frequency (0.5 T) to evaluate its performance. Notably, imaging experiments showed substantial improvement in the SNR, rising from 18.17 to 29.88 (~64%) and from 20.04 to 34.95 (~74%) in the knee and hand, respectively, as shown in Fig. 8. This result confirms that the HINM design remains effective for both low-field and ultrahigh-field MRI.

### Safety analysis of the HINM coil

The SAR distribution of the proposed HINM arrays was assessed on the neck and knee using the Gustav human body model. To assess patient safety, the SAR values were averaged over 10 g of tissue near the skin surface. As shown in Fig. S7, the maximum 10 g SAR values were 0.05 W/kg for the neck and 0.08 W/kg for the knee. These are lower than the safety limits established by the Food and Drug Administration (FDA). Localized energy deposition was primarily observed near the tuning and detuning circuits, attributed to the presence of lumped elements. The HINM array’s design inherently confines the electromagnetic field, which not only enhances imaging performance but also mitigates harmful eddy currents. Coaxial cables used in the array included an outer shield that electrically isolated the inner conductor from surrounding biological tissues. However, the electromagnetic field distribution was intentionally modified by introducing a segmented gap in the outer shield. This design enabled controlled current flow, reducing localized electric field concentrations and improving field uniformity [55]. In continuous shielding, eddy currents can induce power deposition and elevate SAR levels. The introduction of a gap between the shield and inner conductor helps limit these currents, thereby reducing energy loss and minimizing SAR.

Furthermore, the temperature rise at all monitored points was minimal (<1 °C), including at high-current-density areas such as the detuning circuit and central conductor, as shown in Fig. S7. This was lower than 2 °C according to the regulations of the FDA. Thus, the localized, reduced SAR and temperature values confirm the safety of the HINM design, making it a promising option for clinical MRI.

## Discussion

The HINM array represents an important advancement in MRI coil design, integrating self-decoupling technology with a flexible, wireless, modular structure inspired by a “building bricks” concept. This study demonstrates that the HINM array effectively addresses the limitations of the conventional rigid coils by offering enhanced SNR, adaptability to complex anatomical surfaces, and reliable performance under mechanical deformation. Unlike the traditional designs that require precise decoupling adjustments, the modular HINM array can be directly assembled and attached to specific anatomical regions, providing localized signal enhancement with minimal setup complexity. Importantly, the proposed HINM array combined the HIC and metasurface into a novel, unified, flexible, wireless and passive system.

The HINM design addresses several key limitations of the conventional RF coil systems. The traditional rigid coils often struggle to conform to complex anatomical shapes, which can lead to reduced SNR performance in curved or dynamic regions. In contrast, the shielded configuration and modular design of the HINM enabled superior adaptability, ensuring stable electromagnetic performance even under mechanical mechanical deformation. In addition, the HINM design maintained resonance stability under bending up to 90^o^ and stretching up to 80 mm, while stretching at 90 mm increased signal loss and shifted the resonance frequency, resulting in lower SNR, which highlights the limitation of the design. Moreover, the HINM design minimized power dissipation and effectively prevented localized heating. Most importantly, the HINM design is particularly advantageous for imaging dynamic joints such as the knee or wrist, where maintaining consistent coupling and resonance is crucial for achieving high-quality images.

The HINM design also overcomes the decoupling issues faced by existing wireless systems, such as capacitive-loaded or coaxially shielded metamaterials. In addition, the HINM design enabled intrinsic interelement decoupling without additional circuitry, which simplifies the coil construction and reduces mechanical stress. Furthermore, the shielded configuration eliminates the need for additional decoupling circuitry due to minimizing mutual inductive coupling between elements. The coil design is simplified, and dense array configurations are possible without compromising electromagnetic performance. The wearable and dynamic imaging applications benefit greatly from their robustness and adaptability under bending, stretching, and variable positioning. The proposed HINM design can also be used for other anatomical parts of the body, such as the neck (carotid artery) and elbow imaging, as shown in Figs. S8 and S9.

Additionally, the production comparison of the traditional RF coil and the HINM coil is shown in Table S2. The HINM coil eliminates expensive components, making it very low-cost and very light. By adopting a modular approach, the HINM allows for flexible customization, functioning like building blocks to construct tailored coil arrays for diverse imaging needs. Furthermore, the modularity of the HINM array facilitates easy reconfiguration across a variety of anatomies. In this study, we successfully applied the HINM arrays to both the knee and hand. Beyond these cases, the modular “building bricks” structure enables users to rearrange individual elements to match curved or irregular anatomical surfaces, such as the neck or elbow, as shown in the Supplementary Materials. This plug-and-play characteristic enhances clinical versatility, reduces coil preparation time, and supports broader use in patient-specific or motion-prone scenarios. Furthermore, we have implemented representative metasurface [28,33] designs adapted from recent literature and evaluated them at 5 T under identical experimental conditions, as shown in Figs. S1 to S3. Although these structures were originally reported at different field strengths, adapting them to 5 T enables a fair comparison within the same imaging environment. HINM consistently outperformed these alternatives in terms of receiving sensitivity, in vivo SNR, and mechanical flexibility, while offering superior conformability.

Although the HINM coil offers numerous improvements over traditional RF coils at both low and high MRI field strengths, certain challenges persist. Specifically, the SNR gain was most prominent near the surface at both field strengths, with limited impact observed in deeper tissue regions. Furthermore, optimizing the HINM array’s design may improve its utility in advanced imaging scenarios, such as parallel imaging or ultrahigh-field MRI. Future research should also explore broader anatomical validation and integration of the modular “building bricks” into existing MRI platforms.

Nevertheless, the HINM has demonstrated a substantial improvement in SNR when imaging the knee and hand, with clear visualization of cartilage, tendons, and soft tissues. The improved performance is due to the localized magnetic field enhancement of HINM and its ability to maintain consistent signal pickup, even in complex anatomical shapes. The lightweight, flexible, and modular design ensures patient comfort and usability, which is essential for wearable MRI technologies.

This study proposed an HINM coil with self-decoupling characteristics, modularity, wireless, and flexibility, which provides a versatile and scalable solution for wearable MRI. The proposed arrays not only enhance SNR but also adapt to complex anatomies and ensure patient safety, establishing it as an important advancement in MRI coil design. It is noteworthy that the proposed HINM can be used for both low- and high-field MRI, with demonstrable improvements in SNR, which underscores the design adaptability. Future work will focus on further refining the modular “building bricks” design, enhancing performance across a range of anatomical regions, and investigating its integration with next-generation MRI platforms to expand clinical applicability.

## Materials and Methods

### Design of the HINM coil

The HINM coil is constructed from a nonmagnetic coaxial cable with a 5-mm open gap in the shield conductor, which resulted in 3 conductors, as shown in Fig. 2A. The tune and detune circuit were connected at the coil port, which included 2 crossed diodes connected to the shield and a parallel capacitor and inductor connected to the inner conductor, as depicted in Fig. 2B.

Importantly, this circuit did not contribute to interelement decoupling but achieved intrinsically through the shielded configuration. During the RF transmit mode of an MRI system, the receive-only coil must be detuned in order to avoid undesired signals and potential heating. To ensure passive detuning during the transmit mode, a pair of positive-intrinsic-negative (PIN) diodes (UM9989, Microsemi Corporation) was integrated in a cross-diode arrangement at the feed point of each HINM coil element, as shown in Fig. 2B. The forward voltage and the forward current of the passive PIN diodes were 1.0 V and 10 mA, respectively. In this configuration, no external DC bias circuitry was required. Instead, the diodes were passively driven by the incident body-coil transmit field. During the transmit mode, an induced RF voltage from the transmit body coil forward-biases the PIN diodes, effectively shorting the coil at its feed point and detuning it from resonance. In this forward-bias state, each PIN diode exhibits low resistance (typically <1 ohm), allowing the coil to appear as a low-impedance load, thereby preventing current from circulating. A diode becomes reverse-biased due to its circuit configuration during the receive mode, resulting in a high impedance (typically kilo-ohms). The coil becomes resonant in this condition, enabling it to receive MR signals effectively. In addition, a parallel LC tank circuit (formed by a high-*Q* capacitor and inductor) is connected to the inner conductor to adjust the resonance frequency. This configuration enabled passive, automatic detuning without the need for external biasing circuitry, enhancing safety and simplifying integration into existing MRI systems. The cross-diode arrangement provides bidirectional protection and reliable detuning regardless of the polarity of the induced voltages. The resonance frequency performance of the HINM in the tune and detune states is shown in Fig. S10A. The image performance with the tune HINM and the detune HINM in Fig. S10B showed effective switching. The effectiveness of this passive detuning is also demonstrated in Fig. S11.

### Electromagnetic simulations

Electromagnetic simulations of the HINM coil were conducted using CST Microwave Studio to evaluate its current distribution, magnetic field characteristics, and impedance behavior. The unit cell of the proposed HINM coil was simulated with magnetic and electric boundary conditions, as shown in Fig. S12. A 3D model of the HINM coil was constructed, including the inner conductor, outer conductor, and dielectric layer, with dimensions and material properties matching the fabricated prototype, as shown in Fig. 2C. The relative permittivity (ϵr) of PTFE and the conductivity of copper were set according to the cable specifications.

The HINM model was placed on cylindrical and planar phantoms to simulate human anatomy. The simulations focused on 3 key aspects. First, the surface current distribution on the iC, the oCi, and the oCo was analyzed, particularly near the shield gap, where the shielded characteristics enhance current localization. Second, the spatial distribution of the magnetic field in coronal and sagittal planes was evaluated to assess field homogeneity and localization, essential for optimizing signal sensitivity in wearable MRI applications. Finally, the total impedance of the HINM coil was simulated and measured. The resonance performance under varying mechanical deformations was also tested, including bending and stretching, to determine its resonance frequency stability and decoupling performance.

Furthermore, we have conducted electromagnetic simulations to evaluate the worst-case SAR scenarios using a transmit coil along with the HINM arrays. To ensure patient safety, it is necessary to assess the risks associated with the HINM array integrated with an MRI system. We ensured patient safety during the MRI procedure by normalizing all results to meet the SAR limits, including different conditions, such as head, partial body, and whole-body SAR of <3.2, 2 to 10, and 2 W/kg, respectively. Therefore, the standard SAR criteria were utilized to assess the RF safety of the proposed HINM coil, and the computation is outlined as follows [56–58]:

$$\mathrm{SAR}=\frac{1}{M}\int \mathrm{SAR}\left(r\right)\mathrm{dm}=\frac{1}{2M}\int \delta {\left|E\left(r\right)\right|}^{2}\mathrm{dv}$$ (1)

where *M* is the mass of the region for integration over 1 or 10 g. The maximum value of SAR is extracted over the region of 1 or 10 g tissues in the ROI, such as the neck and knee.

### Temperature measurement

To evaluate potential RF-induced heating, we conducted a controlled thermal test using 8 fiber optic temperature probes (S1 to S8) placed at key locations across the HINM array, including the detuning circuit and central coil regions most susceptible to RF-induced heating. These measurements were performed under loaded conditions using a standard cylindrical phantom. A duty-cycle sequence was used with parameters representative of clinical scans: TR = 100 ms, TE = 20 ms, FA = 30°, slice thickness = 5 mm, FOV = 250 mm × 250 mm, and matrix size = 256 × 256. The imaging session lasted 10 min, and the ambient room temperature was maintained at 25 °C. Temperature readings were recorded at 1-min intervals.

### In vitro and in vivo setup

The in vitro and in vivo experiments were conducted on a 5-T MRI scanner (UMR Jupiter 5T, Shanghai United Imaging Healthcare, China) operating at a Larmor frequency of 210.8 MHz. For in vitro experiments, a cylindrical phantom (diameter 100 mm, length 200 mm) filled with a solution of 1.243 g/l NiSO₄·6H₂O and 2.6 g/l NaCl in deionized water (conductivity *σ* = 0.53 S/m) was used.

The HINM coil was positioned around the phantom with a 3-mm gap to ensure consistent electromagnetic coupling. A 2-dimensional (2D) gradient echo (GRE) sequence was applied for the SNR calculation with the following parameters: field of view (FOV) = 250 mm × 250 mm, slice thickness = 5 mm, repetition time (TR) = 100 ms, echo time (TE) = 20 ms, flip angle (FA) = 30°, and matrix size = 256 × 256, while the noise images were obtained by setting the FA to zero. The B_1_^+^ maps were obtained using a dual refocusing echo acquisition mode (DREAM) sequence, with parameters of TR = 1,000 ms, TE1/TE2 = 1.5/4.09 ms, FA = 54.7°, slice thickness = 10 mm, FOV = 300 mm × 300 mm, and matrix size = 64 × 64.

For the in vivo experiments, the imaging scan was performed on healthy volunteers after obtaining informed consent, with study approval from the local ethics committee. The HINM arrays were placed directly on the target anatomical region, such as the anterior knee and dorsal hand surface, with the integration of a knee and commercial flexible array coil. The HINM array functioned as supplemental receive elements positioned adjacent to or slightly underneath the commercial coils to enhance localized signal reception. The high-impedance design of the HINM array ensured intrinsic decoupling, minimizing mutual coupling with the commercial coils, and preserving stable resonance and overall coil sensitivity profiles during imaging. Furthermore, quadrature high-pass birdcage coils were used for transmission. The image quality using the HINM arrays was compared to that using only the commercial coils. The in vivo imaging sequence for the knee included a proton density-weighted fast spin echo (PD-FSE) sequence with parameters TR/TE = 2,880/26.64 ms, FA = 110°, FOV = 160 mm × 160 mm, slice thickness = 3 mm, and matrix size = 432 × 432, and a T2-weighted fast spin echo (T2-FSE) sequence with TR/TE = 5,000/76 ms, FA = 110°, FOV = 160 mm × 160 mm, slice thickness = 3 mm, and matrix size = 432 × 432. We used a 2D GRE sequence to calculate the SNR of the knee with the following parameters: FOV = 200 mm × 200 mm, slice thickness = 5 mm, TR = 200 ms, TE = 20 ms, FA = 30°, and matrix size = 256 × 256; the noise image was obtained by setting the FA to zero. For the hand imaging, a T1-weighted fast spin echo (T1-FSE) sequence was applied with TR/TE = 610/9.98 ms, FA = 110°, and voxel size = 0.53 mm × 0.53 mm × 3 mm. For the SNR calculation, the same 2D GRE sequence was applied with a proper FOV. For vascular imaging, non-contrast-enhanced 3D phase-contrast MR angiography was primarily applied to visualize the vessels with a resolution of 0.5 × 0.5 × 0.5 mm^3^.

To evaluate the low-field performance of the HINM coil, in vivo experiments were conducted on a 0.5-T MRI scanner (0.5T, Marvel Stone Healthcare, China) operating at a Larmor frequency of 21.3 MHz. For the knee imaging, a PD-FSE sequence with the following parameters was applied: TR = 2,000 ms, TE = 25 ms, FA = 90°, FOV = 160 mm × 160 mm, and matrix size = 200 × 200. A T2-FSE sequence was also applied with the following parameters: TR = 3,920 ms, TE = 100 ms, FA = 90°, FOV = 160 mm ×160 mm, and matrix size = 200 × 200. A 2D GRE sequence was applied to calculate the SNR with the following parameters: FOV = 160 mm × 160 mm, slice thickness = 3.5 mm, TR = 140 ms, TE = 5.5 ms, FA = 69°, and matrix size = 200 × 200; the noise image was obtained by setting the FA to zero. For the hand imaging, a T1-FSE sequence was applied with TR/TE = 310/15 ms, FA = 90°, and voxel size = 0.8 mm × 0.8 mm × 3 mm. For the SNR calculation, the same 2D GRE sequence was applied with a proper FOV.

### Data analysis

The acquired data were processed to evaluate the performance of the HINM coils in terms of SNR, B_1_^+^ maps, and resonance stability. The SNR image for the HINM was assessed using the covariance-weighted root-sum-square (Cov-rSOS). In this context, *S* represents the complex signal vector from the HINM, and *Ψ* represents the noise covariance matrix. The SNR was calculated using the formulation described in [59,60]:

$$\mathrm{SNR}=\sqrt{S^{H}\psi^{-1}S}$$ (2)

where *S*^H^ signifies the Hermitian transpose of the signal vector *S*, and *Ψ*^−1^ is the inverse of the noise covariance matrix. This approach ensures an accurate estimation of the effective SNR by properly incorporating interchannel noise correlations, which is especially relevant in multielement and metasurface-assisted MRI systems such as the HINM configuration. Furthermore, all data were processed using MATLAB (MathWorks, Natick, MA, USA), including the import of raw data and SNR computation based on separately acquired signal and noise scans using the Cov-rSOS method. The average SNR values were calculated programmatically in the ROI.

The B_1_^+^ maps were obtained using the vendor-provided DREAM sequence from the MRI system, which was directly generated in digital imaging and communications in medicine (DICOM) files. These were analyzed and visualized in MATLAB. For certain DICOM datasets, MATLAB was also used to read signal intensity along specified directions, and plot functions were applied to assess variations. MATLAB plotting and image processing toolboxes were used for visualizing results, ensuring a clear and reproducible presentation of SNR distributions and B_1_^+^ field maps.

## Ethical Approval

This study was approved by the institutional review boards of Shenzhen Institutes of Advanced Technology of the Chinese Academy of Sciences. Informed consent was obtained before the imaging was implemented.

## Data Availability

Data are available upon reasonable request.
