# Supplementary material for: High-Impedance Nonlinear Metasurface Arrays with Self-Decoupling for Modular and Wearable Magnetic Resonance Imaging
Source: Research (Wash D C). 2025 Dec 22;8:1057. doi: 10.34133/research.1057 (PMC12719561; doi:10.34133/research.1057)
Supplement: Supplementary 1 — Figs. S1 to S12 Tables S1 and S2 [file research.1057.f1.docx]

**Supplementary materials**

To faithfully transplant the NLMM design at 5T as described in the literature [28], we followed its fabrication process: first, we prepared a support with wire grooves using fused deposition molding (FDM) three-dimensional (3D) printing with polylactic acid (PLA) material to position the coil elements; the solenoid part was wound with 0.3 mm enameled copper wire along the support grooves for 2.75 turns to form a micro-solenoid inductive unit; the split-ring was constructed with an open metal ring structure, equipped with an adjustable capacitor at the gap to tune the unit's resonance frequency and paralleled with a pair of bidirectional diodes at its ends for automatic detuning protection during high-power transmission phases; each solenoid paired with its corresponding split-ring formed a nonlinear meta-atom (NMA), and four NMAs were arranged with a predetermined periodicity to compose a NLMM layer; by adjusting the inter-unit spacing and the value of the adjustable capacitor, we performed coarse and fine tuning of the overall resonance response to achieve an array resonance frequency of 210.8 MHz. Finally, during imaging experiments, we optimized the coupling strength and verified the enhancement in reception sensitivity by varying the gap between the NLNM and the receiving surface coil, adhering to the optimal distance of 2 cm as specified in the original text. The photographs of the NLMM design are showed in Fig. S1a.

We strictly adhered to the coaxial shielded circular resonator (CCR) structure reported in the literature [33] for faithful reproduction and optimization at 5T. Specifically, we utilized commercially available RG316 coaxial cable to construct a ring coil with a diameter of approximately 12 cm, uniformly cutting three pairs of inner and outer gaps on opposite sides of the cable to form a multi-gap design that supported structural self-capacitance and suppresses the electric dipole moment effect; PIN diodes were loaded at each inner conductor gap for passive detuning. The coil's resonance frequency, primarily determined by the total length of the cable and the geometric gaps, was finely tuned using a copper foil sleeve with a diameter of approximately 2 mm, which adjusted the effective parallel plate capacitance by covering or exposing the gaps, thereby precisely setting the resonance frequency to 210.8 MHz. To meet the geometric requirements for comparative imaging with different metamaterial coils, we further reshaped the coil into an elliptical structure while maintaining the tuning sleeve position unchanged, ensuring frequency stability and facilitating imaging comparison validation. The photographs of the CCR design are showed in Fig. S1b.


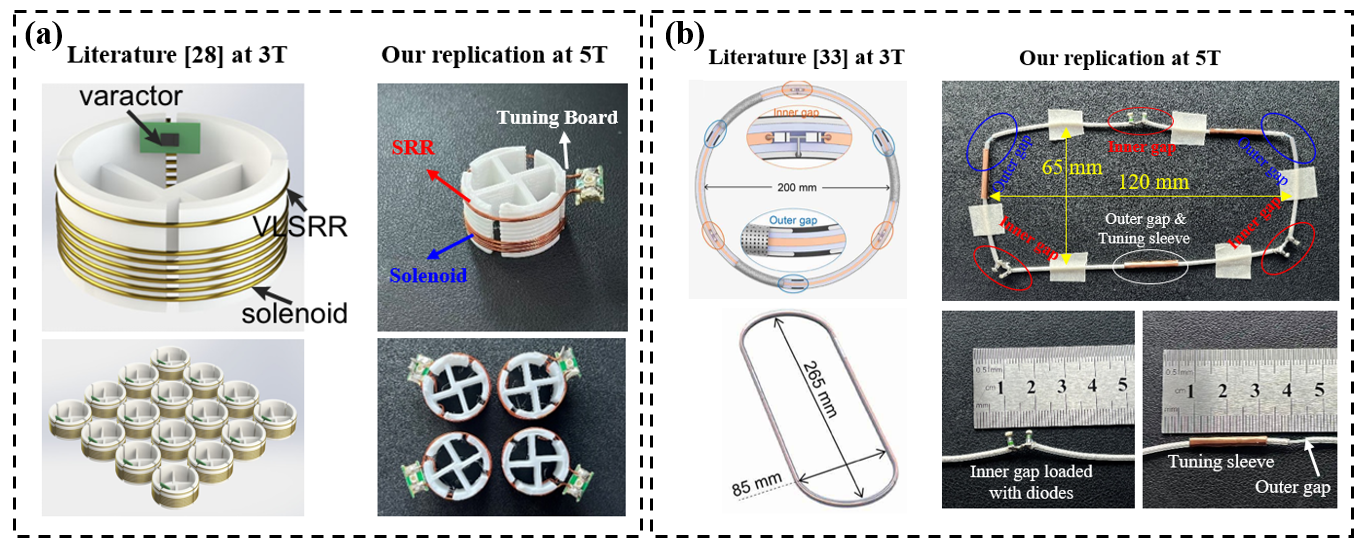


***Fig. S1.*** *The photographs of the NLMM (a) and CCR (b) designs..*

Fig. S2 shows the resonance frequency performance of the prototypes under different stretching. The frequency offsets are listed in the table, from which can be seen that the HINM produced only minimal changes in resonance frequency, while the CCR design showed large changes. And the NLNM cannot be stretched. The different coil prototypes highlight practical differences: the NLMM is rigid and lacks adaptability, the CCR design is flexible but has poor tensile performance, while the HINM is flexible and stretchable. Thus, the aforementioned results demonstrate that HINM outperforms previous designs both in flexibility and stretchability.


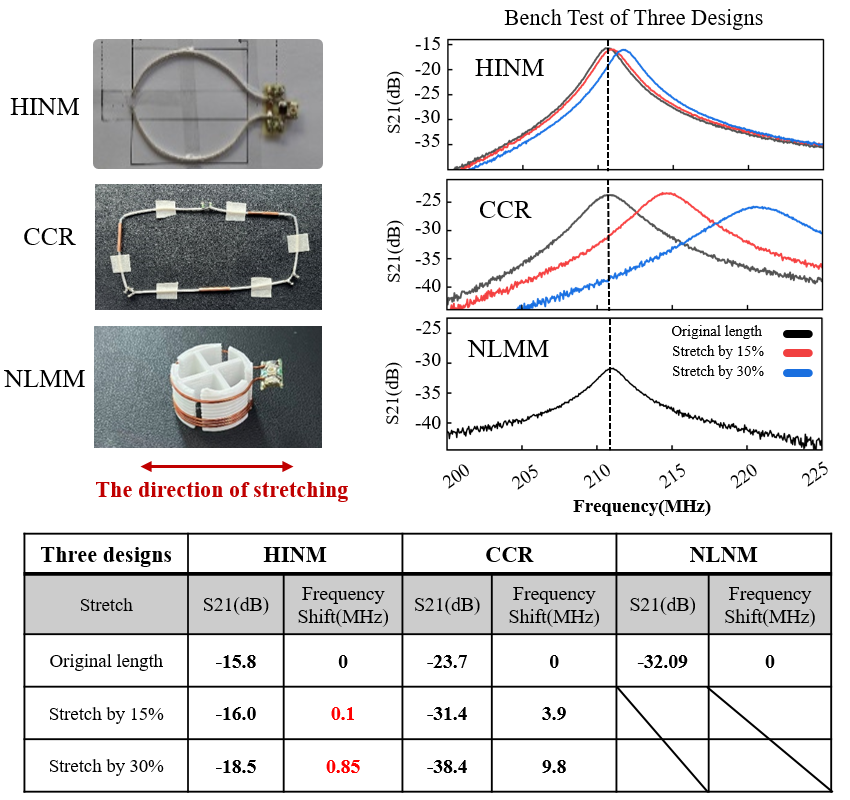


***Fig. S2.*** *Resonance frequency performance of the prototypes under different stretching.*

In addition, Figure 4 presents the in-vivo experiments of the different designs at 5T. Due to the self-decoupling feature, the HINM coil achieved better image uniformity than the NLMM and the CCR, as indicated by the red circle in Fig. S3.


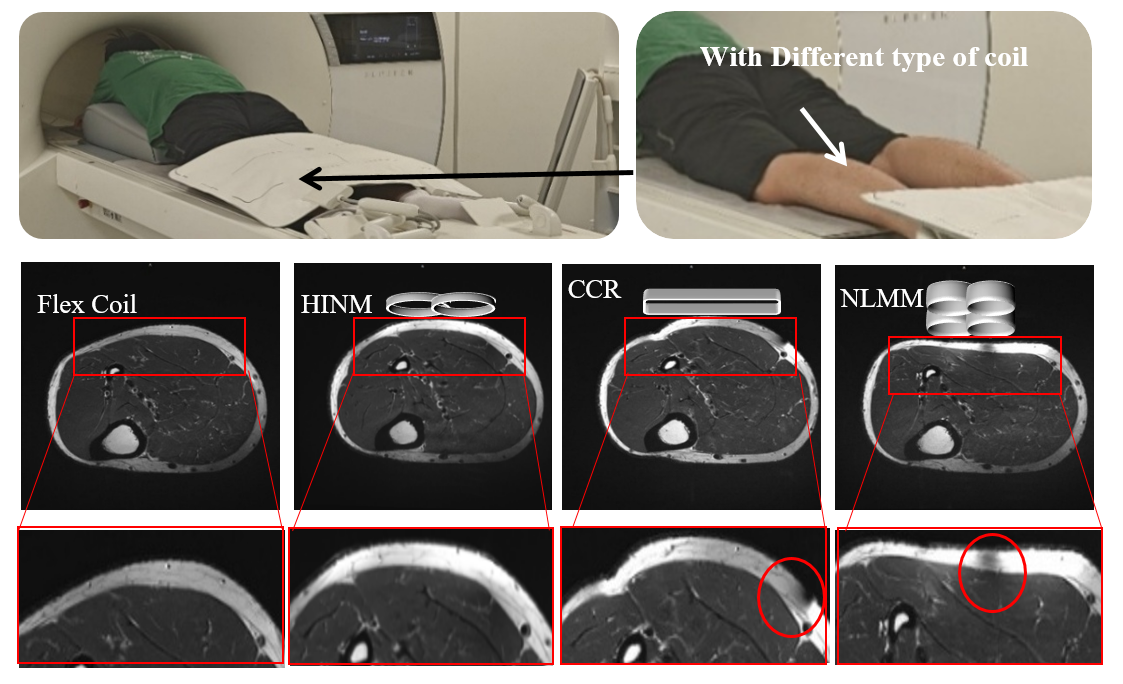


***Fig. S3.*** *In-vivo thigh imaging using various coils (flex, HINM, CCR, and NLMM) at 5T.*

Table S1 shows the comparison of the proposed HINM with the previous start-of-the-art linear metasurface (LM) and nonlinear metasurface (NM) designs.


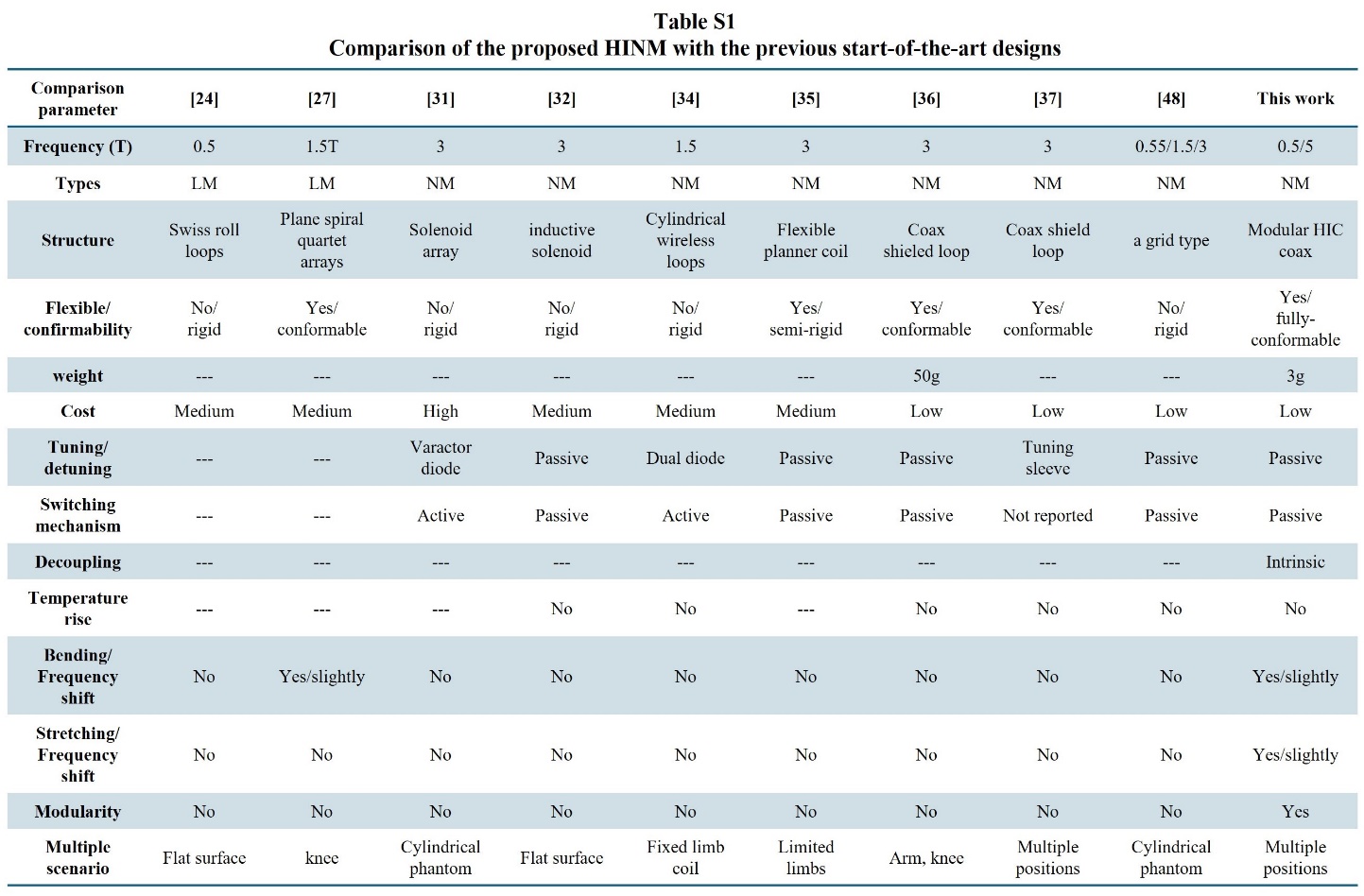


Since the HINM was a resonant structure of wireless and portless, in order to evaluate its transmission characteristics and coupling characteristics, it is necessary to match and tune it according to the wired RF coil. After tuning at 210.8 MHz and matching to 50 Ohm, both the adjacent two HINMs and the overlapping two HINMs demonstrated good decoupling performance, as shown in Fig. S4.


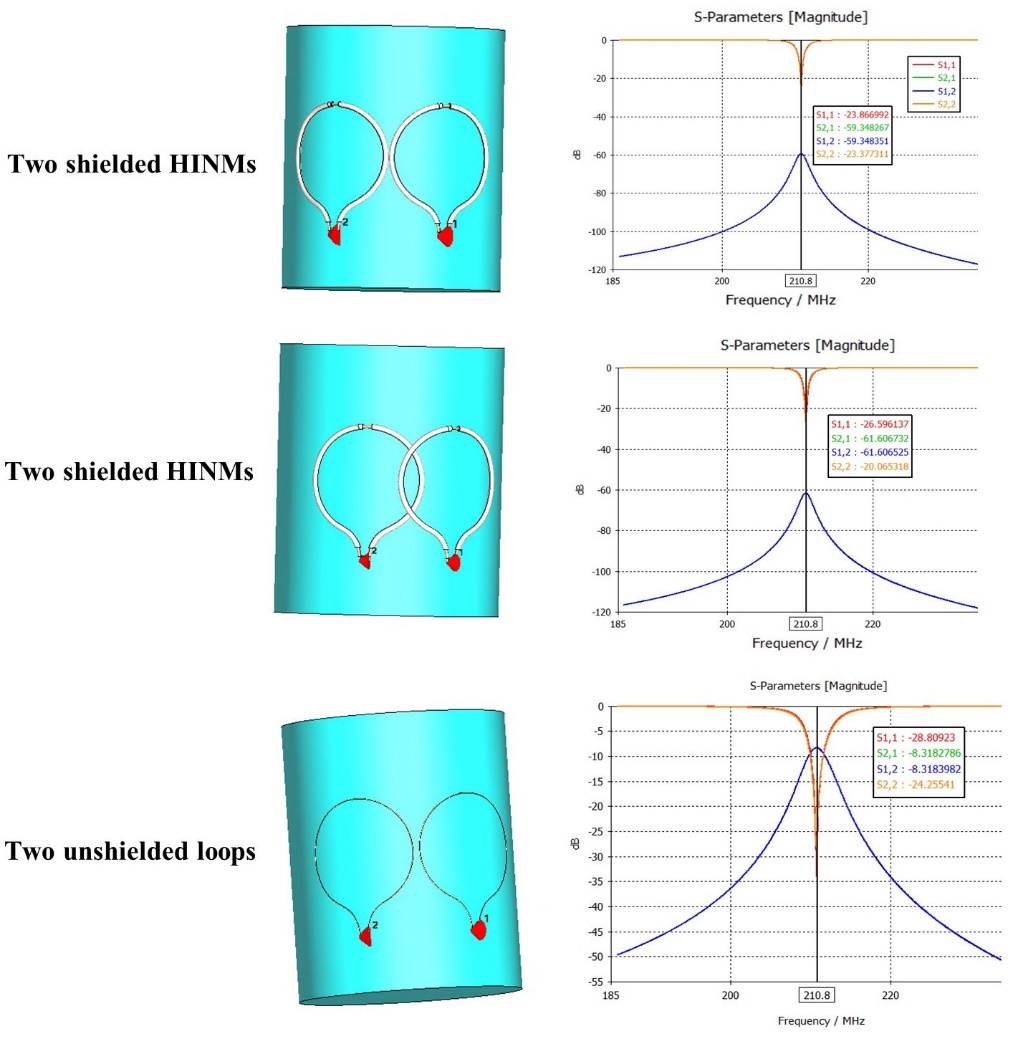


***Fig. S4.*** *The decoupling performance of the shield HINMs and unshielded loops.*

Fig. S5 shows the bending cycle performed up to 1000 times to evaluate the S21 and Q-factor performance.


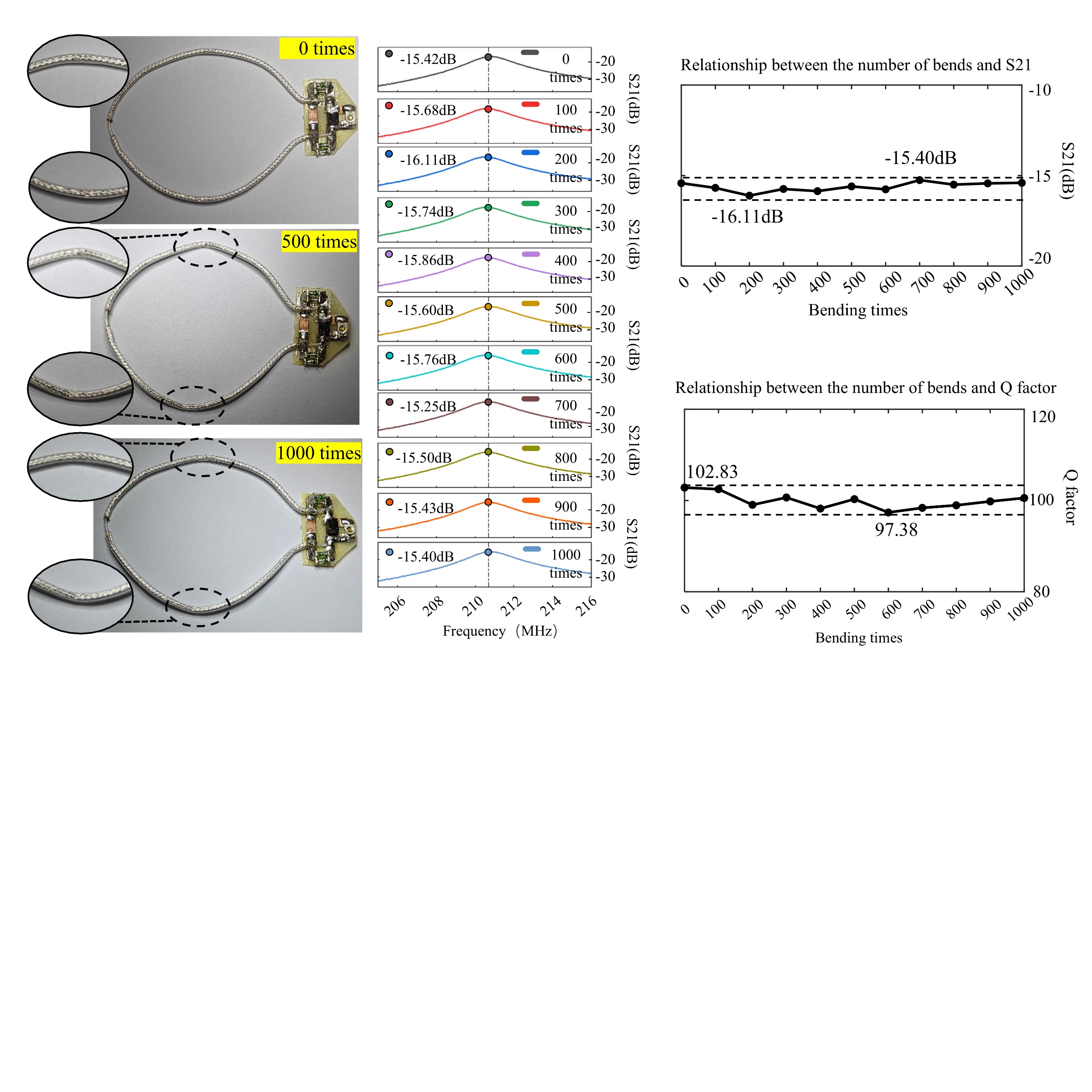

***Fig. S5.*** *Electromagnetic and structural stability of the HINM coil under 1000 cyclic bending tests, showing minimal shifts in resonance frequency, S21, and Q-factor.*

Fig. S6 illustrates the surface current distribution of the HINM between the elements, with a gap of approximately 0 mm.

***
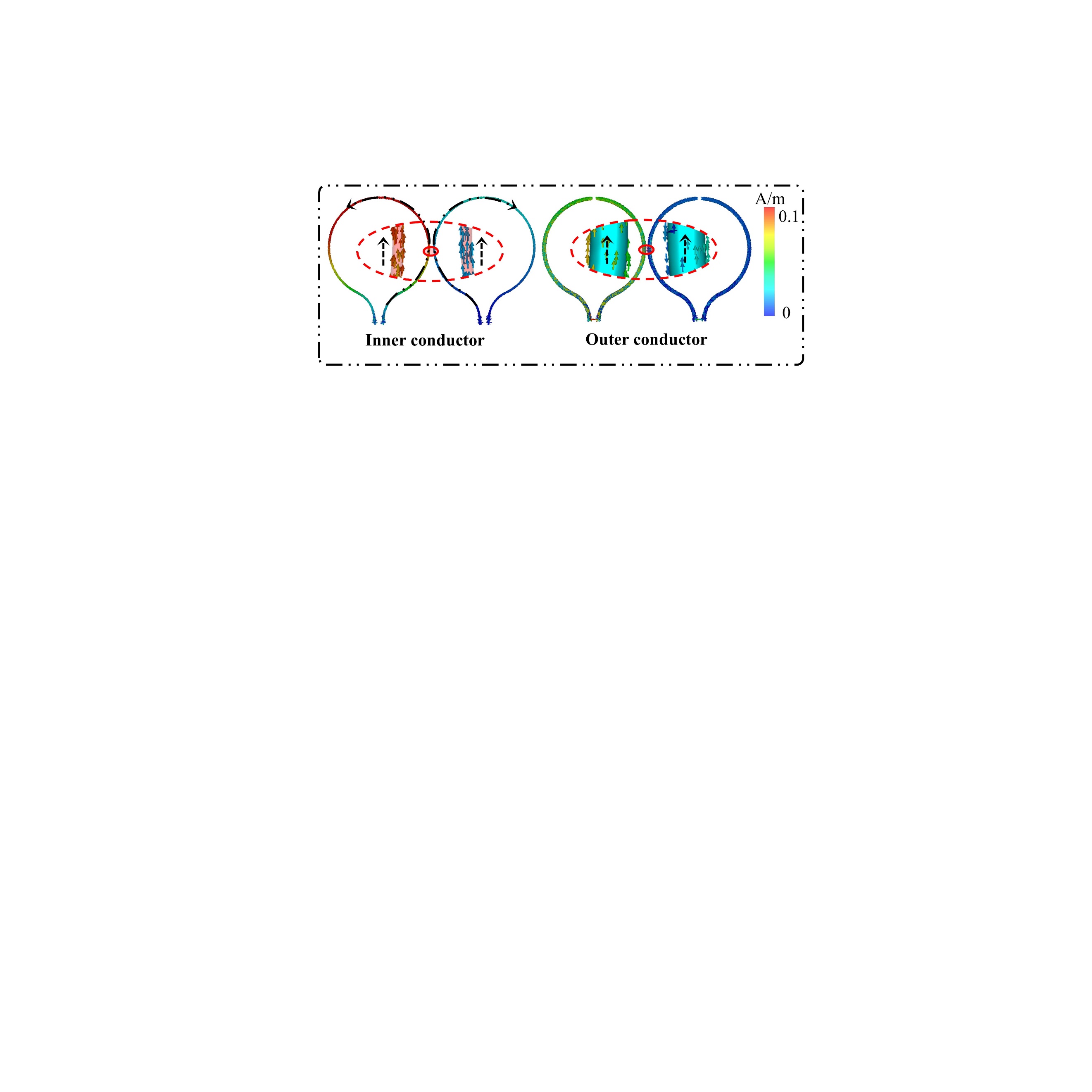
***
***Fig. S6.*** *Current distribution on the inner and outer conductor with approximately 0 mm gap.*

Fig. S7 presents the RF safety analysis and temperature measurement results.


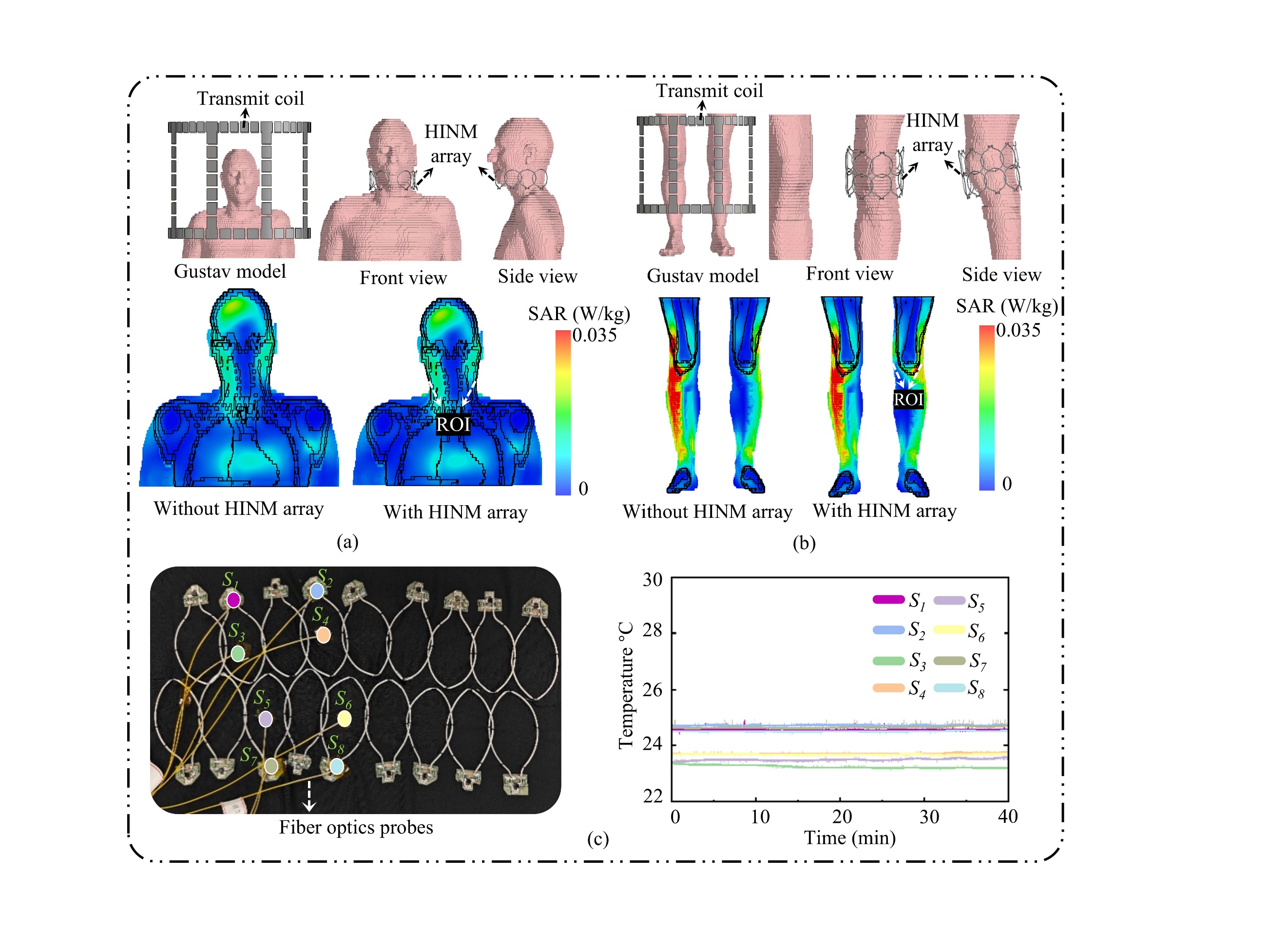

***Fig. S7.*** *SAR distribution and temperature measurement for HINM array. (a–b) Peak 10 g SAR with and without HINM array on torso and legs. (c) temperature rise measured by fiber optics probes placed at different positions of the high-impedance nonlinear metasurface (HINM) array. S1 to S8 indicate the measuring temperature points.*

Building on the successful application of the high-impedance nonlinear metasurface (HINM) array for knee and hand imaging, we further extend the HINM design to various configurations, as illustrated in Fig. S8. These include: (1) arm fully extended, (2) elbow flexed at 90°, and (3) arm stretched to introduce up to 10 mm of displacement. For benchmarking purposes, scans were also conducted using a commercial flexible coil under identical conditions.

All scans were acquired using a T1-weighted fast spin echo (T1_FSE) sequence with the following parameters: TR = 1010 ms, TE = 9.08 ms, flip angle = 88°, slice thickness = 3 mm, bandwidth = 360 Hz, field of view = 200 mm × 160 mm, and matrix size = 580 × 464.

Based on the resulting images, the HINM coil consistently visualized musculoskeletal anatomy clearly in all configurations. Compared to the commercial coil, the HINM array provided more uniform signal distribution and higher tissue contrast, particularly around the elbow joint. Notably, key anatomical structures such as the cortical bone (humerus and ulna), joint space, surrounding muscles, and fat layers were better delineated. The improvement of image quality and signal strength was obvious.

Importantly, the HINM coil maintained image quality and SNR consistency even under mechanical deformation (e.g., 10 mm stretching or 90° flexion). This robustness, combined with its mechanical adaptability, highlights its potential for applications in dynamic joint imaging or anatomically variable regions where flexibility and patient comfort are critical.


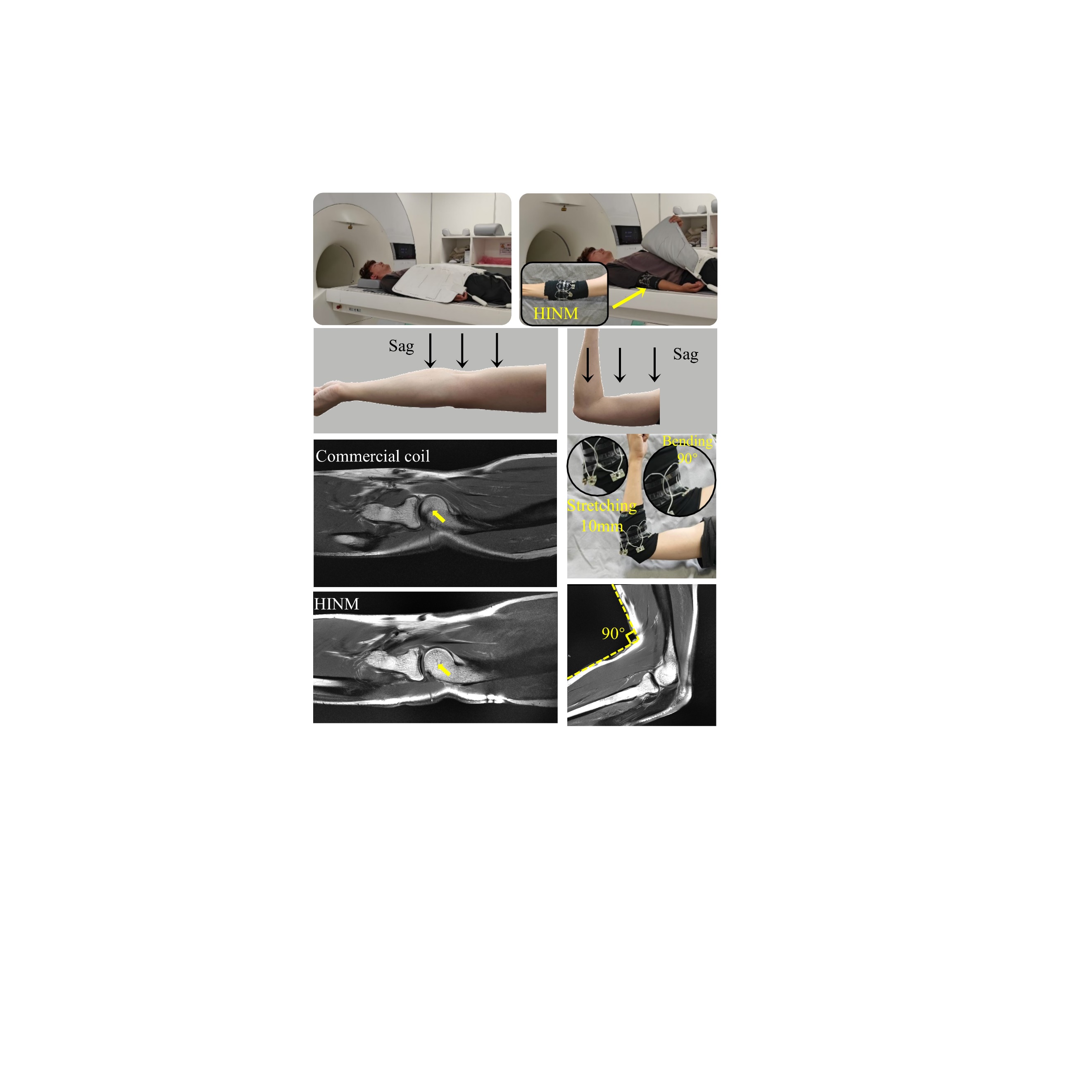

***Fig. S8.*** *MRI comparison using commercial flexible coil and HINM array. HINM shows improved image clarity with HINM under flexible conditions, demonstrated through subject positioning, arm deformation, and corresponding MRI scans.*

In addition, we also extended the application to carotid artery imaging by designing an 8-channel HINM array tailored for the anatomical curvature of the human neck. The carotid artery, located in the cervical region, presents a unique imaging challenge due to its proximity to highly curved anatomical structures and dynamic movements. Traditional coils often struggle with maintaining optimal signal reception in such regions, leading to potential compromises in image quality. Given the demonstrated robustness of the HINM array under bending and stretching conditions, we employed a similar flexible and high-impedance design for the carotid artery coil. The inherent mechanical resilience of the HINM ensured minimal performance degradation due to shape deformation, making it particularly suitable for vascular imaging in the neck. We utilized a three-dimensional (3D) time-of-flight (TOF) sequence to perform non-contrast-enhanced magnetic resonance angiography (MRA) of the carotid arteries at 5T, of which parameters are: TR=24.9 mm, TE=5.3 ms, flip angle=15^o^, FOV = 200 mm × 230 mm, slice thickness = 0.3 mm, matrix size = 403 × 464, and bandwidth=210 Hz/pixel. The resulting images provided high spatial resolution and excellent visualization of vascular structures, especially at the carotid bifurcation, where detailed anatomical features were clearly distinguishable, as shown in Fig. S9. The results

demonstrate that the 8-channel HINM carotid coil offers significant advantages in SNR and image clarity, particularly in depicting complex bifurcations and small vascular structures. By leveraging the modular and flexible properties of the HINM, this design offers a promising alternative to conventional carotid imaging techniques.

***
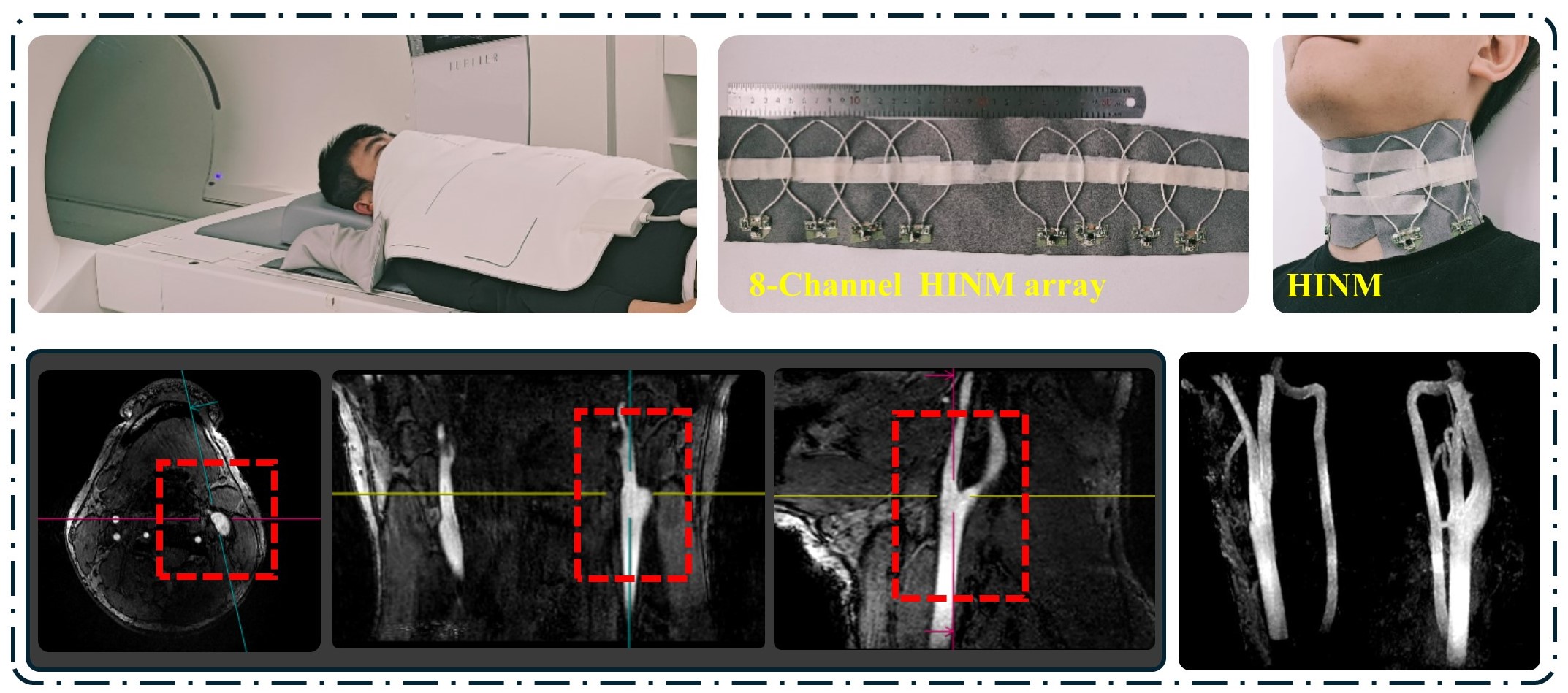
***

***Fig. S9.*** *The carotid artery imaging with a resolution of 0.5 × 0.5 × 0.3 mm^3^ at 5T using the commercial flexible coil with an 8-channel HINM array.*

Table S2 demonstrates the production comparison of the traditional radio frequency (RF) coil and the HINM coil. The traditional RF coils often include non-electromagnetic components, such as adapters, cable traps, feeding boards, and decoupling pre-amplifiers, increasing their bulk and cost. Alternatively, the HINM coil had eliminated expensive components, making it very low-cost and very light.

***Table. S2.*** *The production comparison of the traditional RF coil and the HINM coil.*

| Type | Tuning and matching circuits | adapters | cable traps | feeding boards | pre-amplifiers | Weight  (g) | Cost (USD) | | |
| --- | --- | --- | --- | --- | --- | --- | --- | --- | --- |
| traditional RF coil with N channels | N | N | N | N | N | 100 × N | | 230 × N |  |
| HINM coil with  N elements | N | 0 | 0 | 0 | 0 | 3 × N | | 5 × N |  |

The resonance frequency performance of the HINM in the tune and detune states is showed in Fig. S10a. Fig. S10b further compares three cases of the B_1_^+^ field using a DREAM sequence with a flip angle of 54.7°: (i) commercial flexible coil only, (ii) with tune HINM, and (iii) with detune HINM. It is clearly observed from the figure that the HINM coil can significantly enhance the receive field strength while having minimal impact on the transmit field. If the PIN didoes were always in the state of forward bias and cannot be reverse biased, the B_1_^+^ field would be decreased and black holes would be produced. Only when the PIN diodes were working properly, the B_1_^+^ field and the uniformity of the image would not be affected.


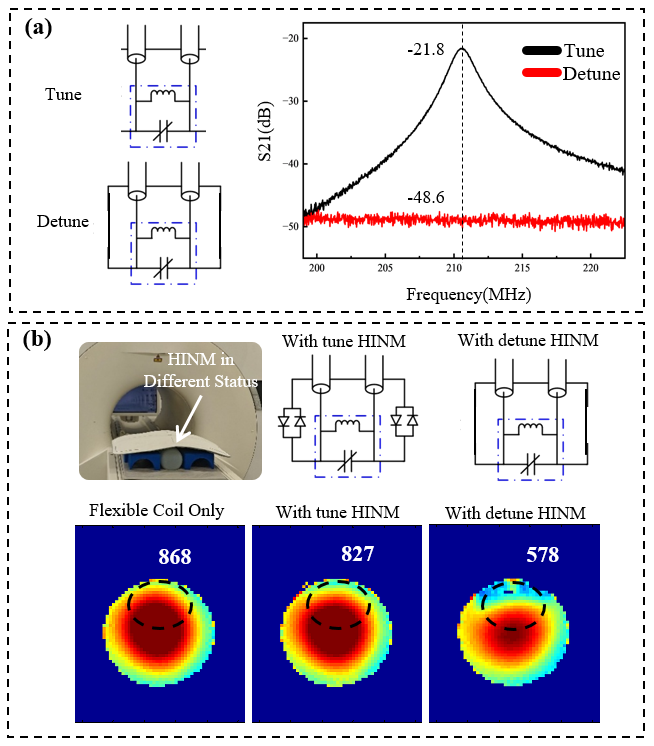


***Fig. S10.*** *Experimental characterization of the PIN diode switching in the HINM coil:* *(a) S21 response confirming tune-to-detune transition; (b) The B_1_^+^ field maps using the flexible coil only, in combination with the tune HINM and the detune HINM.*

The effectiveness of this passive detuning using the DREAM sequence with different reference voltages is demonstrated in Figure S11. The reference voltage was calibrated as 50 V, which indicated that a pulse with a duration of 0.3 ms can turn the flip angle to 54.7^o^ in the DREAM sequence. Based on the definition of the MRI system, the signal strength of 1000 is 5.875 μT when using the Dream sequence for testing. In this case, a strong B_1_^+^ field of 5.10 uT was generated in the transmit state. When the reference voltage was set to 12.5 V, the B_1_^+^ field was markedly reduced to 1.50 μT. The difference in the transmission magnetic field between the RF coil without HINM and the RF coil with HINM was within 10%. These results demonstrate that the diode-based switching remained effective across a wide range of input powers. This ensures that the detuning mechanism still functions even under reduced drive conditions in different clinical pulse sequences.


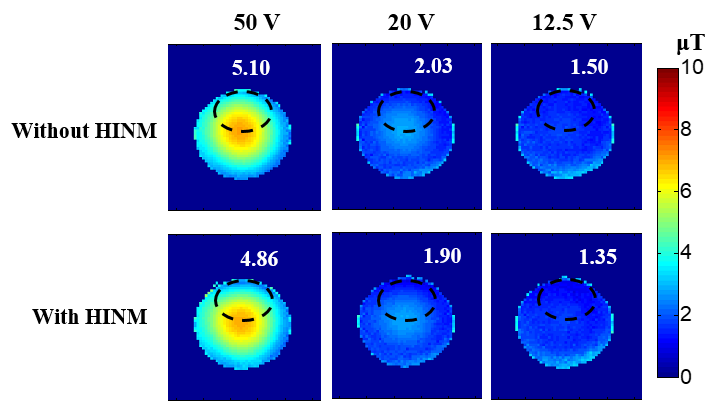

***Fig. S11.*** *The B_1_^+^ field maps using the flexible coil only in combination without and with the tune HINM at different transmit voltages.*

We emphasize that diode switching does not rely solely on the DREAM mapping pulses. In standard clinical imaging, where 90° and 180° pulses of shorter duration are routinely used, the corresponding B_1_⁺ amplitudes fall within the 10–20 μT range. These higher amplitudes provide ample forward bias to the PIN diodes, ensuring reliable switching into the detuned state. Thus, while DREAM operates at deliberately low B_1_⁺ levels, the diodes were experimentally verified to detune under transmit conditions, and under routine clinical pulse sequences, the switching margin is even more robust.

HINM unit cells were simulated by applying periodic boundary conditions to simulate an infinite array, as shown in Fig. S12. Specifically, electric (PEC) boundary conditions were applied to the x-axis faces, while magnetic (PMC) boundary conditions were set on the z-axis faces. Wave ports were positioned on the faces normal to the y-axis, enabling the excitation of magnetic resonance through normally incident plane waves, with magnetic fields oriented along the z-axis.


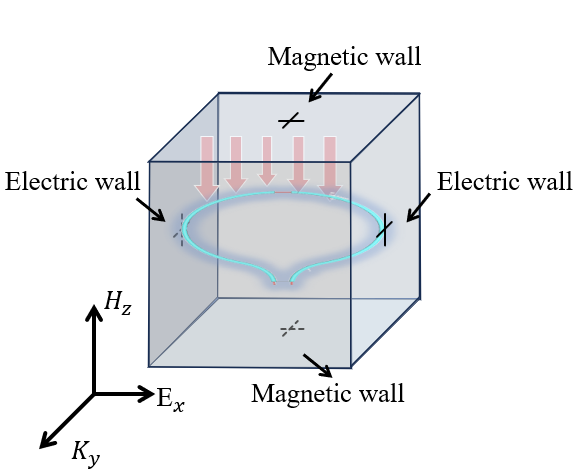


***Fig. S12.*** *Boundary condition of the HINM unit cell.*
